# Recent Development in the Processing, Properties, and Applications of Epoxy-Based Natural Fiber Polymer Biocomposites

**DOI:** 10.3390/polym15010145

**Published:** 2022-12-28

**Authors:** Raed B. Alsuwait, Miloud Souiyah, Ibrahim Momohjimoh, Saheed Adewale Ganiyu, Azeez Oladipupo Bakare

**Affiliations:** 1 Department of Mechanical Engineering, College of Engineering, University of Hafr AlBatin, P.O. Box 1803, Hafr AlBatin 39524, Saudi Arabia; 2 Department of Mechanical Engineering Technology, Applied College, University of Hafr AlBatin, P.O. Box 1803, Hafr AlBatin 39524, Saudi Arabia; 3 Department of Chemistry, College of Chemicals and Materials, King Fahd University of Petroleum and Minerals, P.O. Box 5026, Dhahran 31261, Saudi Arabia; 4 Department of Nondestructive Evaluation, Applied College, University of Hafr AlBatin, P.O. Box 1803, Hafr AlBatin 39524, Saudi Arabia

**Keywords:** biocomposites, epoxy, natural fibers, biodegradable, mechanical properties

## Abstract

Growing environmental concerns have increased the scientific interest in the utilization of natural fibers for the development of epoxy biocomposite materials. The incorporation of one or more fibers in the production of hybrid epoxy polymer composites has been a subject of discussion. It is interesting to acknowledge that natural/synthetic fiber hybridized epoxy composites have superior properties over natural/natural fiber hybridized epoxy composites. Significant efforts have been devoted to the improvement of natural fiber surface modifications to promote bonding with the epoxy matrix. However, to achieve sufficient surface modification without destroying the natural fibers, optimization of treatment parameters such as the concentration of the treatment solution and treatment time is highly necessary. Synthetic and treated natural fiber hybridization in an epoxy matrix is expected to produce biocomposites with appreciable biodegradability and superior mechanical properties by manipulating the fiber/matrix interfacial bonding. This paper presents a review of studies on the processing of epoxy natural fiber composites, mechanical properties, physical properties such as density and water absorption, thermal properties, biodegradability study, nondestructive examination, morphological characterizations, and applications of epoxy-based natural fiber biocomposites. Other aspects, including a review of variables that enhance the mechanical and functional performance of epoxy/natural fibers composites while also increasing the biodegradability of the composite material for environmental sustainability, were presented. The future research focus was elucidated. It is hoped that this review will stimulate and refocus research efforts toward advancing the manufacture of epoxy/natural fiber composites to meet the growing demand for biocomposite materials in the global world.

## 1. Introduction

The manufacturing industry employs various materials, including pure metals, alloys, and composites in their numerous operations. The demerits of pure metals and alloys have shifted the attention of industrialists and scientists to composites to satisfy the expectations of modern materials’ requirements in the current production industry [1,2]. A composite, by definition, consists of two or more materials, of which one is called a matrix and the others are reinforcement, and these have exciting properties [3].

The high glass transition temperature, high modulus, excellent adhesion to a wide range of fibers, and chemical resistance of epoxy resins have made it versatile as a structural thermoset resin. However, the inability of the epoxy resins to be recycled, reprocessed, or dispersed because of their permanent cross-linked structures makes them environmentally unfriendly as compared to their thermoplastics counterparts [4,5,6,7]. Resolving the environmental issue orchestrated by thermoset materials is an important area of concern, and the production of biodegradable polymers and composites could provide a way forward. Thus, the reinforcement of biopolymers or synthetic polymers with natural fibers instead of synthetic or glass fibers in polymer composites is a viable alternative to environmental remediation [8,9,10,11,12]. Biocomposites, as widely defined, refer to composite materials fabricated from natural or biofibers and petroleum-derived non-biodegradable resins [13]. Any biocomposite produced from plant or crop origin, which is likely to be environmentally friendly, is described as a green composite [14]. It is important to state that the production of 100% biobased materials as a replacement for petroleum-based products is economically unviable. However, a combination of petroleum and biobased resources to produce cost-effective materials with extensive applications is more likely to be a viable solution.

Although researchers and scientists have reported exciting mechanical and functional properties of epoxy-based natural fiber composites, the inherent polar and hydrophilic characteristics of natural fibers and the hydrophobic nature of epoxy resins have created impediments in amalgamation resulting in non-uniform distribution of the fiber in the matrix. Consequently, the efficiency of the developed composite is impaired [15]. This is the profound challenge of epoxy-based natural fiber composites. Other issues associated with natural fiber polymer composites include the restricted processing temperature (less than 200 °C), high moisture absorption, the presence of voids at the interface between the fiber and the matrix, and low dimensional stability [16]. To ameliorate these difficulties associated with natural fiber polymer composites, the treatment of natural fibers before their incorporation into the polymer matrix is crucial. Broadly, natural fiber treatment is categorized into two types: Chemical treatment and surface treatment [1]. Chemical treatment is purposefully designed to reduce the hydrophilic nature of the fibers and this includes alkali treatment, acetic acid treatment, silane treatment, benzoyl peroxide treatment, potassium permanganate treatment, stearic acid treatment, seawater treatment, cellulose powder treatment, polymer coating treatment, bleaching treatment, graft copolymerization, and isocyanate treatment [1,17,18]. Surface treatment of natural fibers is aimed at modifying the fiber surface and equally increases the fiber strength leading to enhancement in adhesion mechanisms between the fiber surface and the polymer matrix. Surface treatment includes plasma treatment, vacuum ultraviolet irradiation treatment, ozone treatment, corona treatment, γ-ray treatment, and laser treatment [1]. Among the chemical treatment methods, alkali (NaOH) treatment is the most common as the treatment is simple, effective, economical, and readily scalable [19]. In this treatment method, two parameters (concentration of sodium hydroxide and the treatment time) are most varied to optimize the treatment conditions.

Researchers have opined that hybridization is a possible solution to the limitations of natural fibers as that would reduce the utilization of synthetic fibers and their attendant environmental consequences. By definition, hybridization is the inclusion of two fibers, either natural fiber, synthetic fiber, or a combination of both fibers [20]. The properties of epoxy natural fiber composites rely on the nature of the constituent elements, their compositions, and the production technique employed in the manufacturing of the composites. Common production technology used in the manufacturing of epoxy-based natural fiber biocomposites includes hand lay-up [7,21,22,23], compression molding [24], sheet molding [25], filament winding [26], and vacuum infusion [27]. Ramasamy et al. [22] prepared epoxy containing synthetic Kevlar fiber and kenaf natural fiber using hand lay-up techniques. The mechanical properties of epoxy were reported to have improved with the inclusion of hybrid fibers. Ankit et al. [28] fabricated epoxy containing luffa fiber and nano clay using the compression molding technique. The composition of nano clay varied, and the maximum mechanical properties were achieved at 2 wt% nano clay. As the nano clay content increased beyond 2 wt%, the mechanical properties of the hybrid reduced, and this was attributed to the clustering of nano clay at higher concentrations due to their high density and Brownian motion. The production of epoxy-containing treated alfa fiber through the hand lay-up technique was investigated by Helaili et al. [8]. In their investigation, the epoxy was filled with 10 vol% content and the elastic modulus of the epoxy was increased by 24.16%. Iliyasu et al. [29] studied the effect of deleb palm fiber parameters on the physical and mechanical properties of epoxy composite using the response surface methodology. Here, epoxy resin was reinforced with sodium hydroxide-treated deleb palm fiber, and the composites were prepared using the hand lay-up technique. The optimized two parameters include reinforcement weight compositions (30–40 wt%) and the fiber length (1–3 mm). The results revealed that a fiber reinforcement composition of 38.3 wt% and a fiber length of 3 mm produced the optimum mechanical and physical properties. Ouchte et al. [30] studied the effect of thermal treatment on the mechanical properties of jute/epoxy composites prepared by the hand lay-up technique. The samples after fabrication were thermally treated at different temperatures (25, 40, 60, 80, and 100 °C), and their mechanical properties such as tensile strength and hardness were determined. It was revealed that the jute/epoxy composite treated at 80 °C has the maximum mechanical tensile strength and hardness compared to others and this is attributed to the improvement in the interfacial adhesion between the fiber and the epoxy matrix. In other reports, Reddy et al. [31] and Nigrawal et al. [32] explained the improvement in mechanical and electrical properties of jute/epoxy hybrid composites after sodium hydroxide treatment of the fiber. In their study, jute fiber was treated with 10% NaOH solution for 5 h and 15% NaOH solution for 48 h, respectively. The mechanical and electrical properties of the composites improved significantly, and this was again attributed to the increase in the interfacial adhesion between the fiber and the epoxy matrix.

Arumugam et al. [33] conducted a study on the mechanical, thermal, and electrical performances of sodium hydroxide-treated rice husks and sawdust epoxy biocomposites fabricated by the hand lay-up technique. The mechanical tensile strength of 7.5 MPa and 7.1 MPa were achieved on epoxy containing 50 wt%-treated rice husk and 50 wt% sawdust, respectively. The thermal resistivity of rice husk/epoxy composite and saw dust epoxy composites were 0.1 M^2^k/w and 0.08 M^2^k/w, respectively. Regarding the electrical surface resistivity of the composite, values of 1.27 × 10^10^ Ω/m^2^ and 7.29 × 10^9^ Ω/m^2^ were reported for epoxy/rice husk and epoxy saw dust biocomposites, respectively. The decrease in thermal and electrical conductivity of epoxy with natural fiber inclusion is attributed to the fact that natural fibers have a porous structure that enables them to entrap air within their molecular structure [10,34,35,36,37,38]. The acoustic performance of epoxy-containing rice husk and sawdust was equally evaluated, and the sound absorption of epoxy was improved with rice and sawdust incorporation. This is because the sound absorption characteristic is related to the internal structure of the natural fibers. Thus, a hollow tubular structure with multiple minute pores helps to abate the transmission of vibrations caused by mechanical disposition over the entire material.

It is crucial to acknowledge the fact that some review works [39,40] have been performed on epoxy natural fiber polymer composites, and these previous efforts are centered on the processing, properties, and application of natural fiber epoxy polymer composites. However, a review paper detailing the uses of nondestructive testing, thermal stability, and biodegradability study of epoxy natural fiber polymer composites is lacking. Therefore, the current effort is garnered toward the processing, properties, thermal stability, nondestructive technique, applications, and biodegradability studies of epoxy-based natural fiber biocomposite materials. Natural fiber treatment before their inclusion in an epoxy matrix, volume concentration, and the control of processing parameters all contribute to the overall properties of epoxy/natural fibers polymer composites.

## 2. Processing of Epoxy-Based Natural Fiber Polymer Composite Materials

The processing and fabrication of epoxy-based natural fibers are paramount as the properties of composites are conditioned on the constituent elements of the composites, production technology, and the techniques employed in organizing the fibers [4,41,42,43,44]. In addition to high strength and stiffness at a low density, composite materials exhibit interesting high corrosion and chemical resistance combined with low cost [25]. However, these awesome properties are not only determined by the incorporated fibers but the matrix also remarkable an important role, as well as the interaction between the matrix and the reinforcing fiber components of the composites. An epoxy matrix is characterized by high hardness, wear resistance, and static and dynamic impact strength, and is usually utilized to achieve the properties of fibers, reduce the surface damage of the fiber, and inhibit the propagation of cracks in the composites [45].

One major interesting aspect of lignocellulosic natural fibers is their positive environmental impact and excellent acoustic insulating properties, although their polar and hydrophilic nature impairs their uniform distributions in the non-polar epoxy matrix, leading to poor compounding and consequently affects the interesting properties of the composites [7,45,46,47]. Natural fibers generally could be termed as a composite primarily comprising cellulose fibrils incorporated into a lignin matrix [12]. The main components of lignocellulosic fibers (natural fibers) are cellulose, hemicellulose, lignin, pectin, and waxes [11]. Lignin availability in the natural fibers could lead to poor bonding between the fiber and the matrix and eventually result in the removal of the layers [8]. To ameliorate this bonding issue, natural fiber treatment is paramount. There are many methods of fiber treatment before their use as reinforcement in epoxy-based composites. The techniques for fiber treatment include chemical, mechanical, and thermal methods [48,49,50,51,52,53]; however, among the treatment methods, chemical treatment (sodium hydroxide) is the most common, economical, and industrially scalable fiber treatment widely reported.

Ranjan et al. [11] studied the comparative analysis of sodium hydroxide and hot water treatment of rice husks fiber. It was discovered that sodium hydroxide (2% NaOH)-treated rice husks in biodegradable epoxy containing 20 wt% rice husks exhibited the highest mechanical tensile and impact strength compared to hot water and their untreated rice husk epoxy composite counterparts. It is indeed important to note that boiling natural fibers in sodium hydroxide could lead to significant degeneration as the oxidizing agent attacks all the other components of the fiber, leaving only cellulose or fibril [54]. In another report, Rajeshkumar et al. [55] explored the influence of sodium hydroxide treatment concentrations (5, 10, 15, and 20%) on the mechanical properties of epoxy-containing phoenix sp. plant fibers for 1 h. It was revealed that a 15% sodium-hydroxide-treated phoenix sp./epoxy composite had the highest mechanical properties. It was therefore concluded that below a concentration of 15%, the fiber was inadequately treated, while beyond a 15% NaOH concentration, damage to the fiber (making it less dense and brittle [56]) occurred, which impaired the mechanical performances of the composites. For chemical treatment, time and concentration are the two major parameters that need to be optimized to ensure that adequate treatment of the fiber is achieved.

Currently, different manufacturing techniques have been used for the production of epoxy-based natural fiber composites [57]. This includes hand lay-up [58,59,60,61,62,63,64], compression molding [65,66,67,68,69,70], sheet molding [71], cold press [72], filament winding [73], and vacuum infusion [74], which are the most common techniques that have been reported in the production of epoxy-based natural fiber composites.

**Figure 1:**
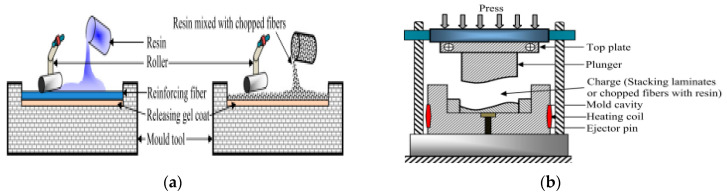

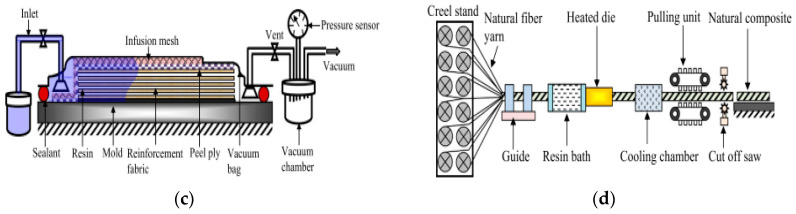
Epoxy manufacturing techniques: (**a**) Hand lay-up, (**b**) compression molding, (**c**) vacuum-assisted resin transfer molding, and (**d**) pultrusion [16] (Reprinted/adapted with permission from Elsevier Ref.5457180037607).

**The hand lay-up technique** is the most popular method to produce biocomposite epoxy both in small- and large-scale manufacturing (Figure 1a). The equipment is simple, very economical, and applicable to a variety of products fabrication. Usually, the mold is coated with gel to facilitate the removal of the composite. After coating, the fibers are manually placed in the mold and then the resin containing the hardener is applied. Uniform distribution of the resin mixture is achieved with the help of a roller to ensure that entrapped air is removed from the composite matt. Enough time is allowed for curing to take place and then the composite is removed from the mold for further processing [75]. Although this technique can be used to produce large-scale precision components, it requires temperature control to avoid the decomposition of the fibers used to produce the components.

**Compression molding** is a common processing technique used for epoxy/natural fiber component manufacturing because of its simplicity and versatility. Here, the short fiber is pre-mixed into the epoxy resin before the application of heat or pressure to achieve full impregnation and shape. In this method, a mixture of resin and fiber is placed in the mold cavity with the required shape component, and then the upper part is placed to close the mold with the subsequent application of pressure to force the premix to compact and take the shape of the mold [76]. It is a high-volume and high-pressure technique used for the manufacturing of complex and high-strength epoxy biocomposites (Figure 1b). The technique is suitable for the production of complex part geometries with different fiber orientations; however, the size of the composite yield is limited by the size of the presses. Moreover, the processing method is labor intensive as the processor is vulnerable to liquid resins and their volatile emulsions [77].

**Vacuum-assisted resin transfer molding technique** is a low-temperature and low-pressure technique that is capable of producing high-performance structures on a large scale [78] (Figure 1c). Usually, the natural fiber is placed inside the mold and then the liquid resin is pumped and transferred inside the mold through the fiber. The extra resin is recovered from the other end of the mold. Finally, the components are ejected from the mold after sufficient time is allowed for curing. This technique is good for the production of large parts; however, due to the lower applied pressure and inherent loftiness of most lignocellulosic fibers, low-volume fiber fraction is required, and this makes the technique unsuitable for the large volume fraction of natural fiber composite development [77].

**The pultrusion technique** is an automated process used to produce continuous constant cross-section composites. It is reported to be suitable for manufacturing unidirectional natural fiber epoxy composites. It is used for the fabrication of a fixed-type profile based on the required shape (Figure 1d). Here, continuous fiber from the roving is pulled and processed through the resin bath, and thereafter, the melted resin is infused with the fiber and shaped to the required shape component [79]. As the method relies on the pulling of continuous roving through a resin bath/impregnator, care must be taken during production using natural fibers due to their yarn’s low pull strength. As a result, pull speeds are usually limited to 100–200 mm/min, which is low when compared to the pull speeds used during glass fiber/epoxy processing [80].

The choice of the above manufacturing methods is dictated by various factors comprising the component material properties, size, and geometry of the composite, and the cost involved in the processing of the composite. Rangappa et al. [81] prepared epoxy hybrid composites reinforced with chicken feather fiber and Ceiba pentandra bark fibers. Firstly, both natural fibers were mixed with bio epoxy and stirred continuously on a magnetic stirrer hot plate at 60 °C for 30 min. The stoichiometric amount (epoxy to curing ratio: 9:3.33) was added to the mixture at room temperature and stirred for 2–3 min. The solution was then poured on a casting mold to cure overnight. Finally, after casting, the hybrid composite was prepared by compression molding and cured for 24 h. Post-curing of the hybrid composites was performed at 80 °C for 24 h in an oven, and composites of dimension 200 × 200 × 3 mm were produced. A hand lay-up approach in the fabrication of hybridized jute fiber, Grewia optiva fiber, and glass fiber in epoxy matrix composites has been presented [15]. The glass fiber and natural fibers were mixed thoroughly and then poured on a glass mold with dimensions of 500 × 300 × 4 mm already containing an amount of epoxy resin. The remaining resin was then spread over the mat, and a 15 kg load was held above the sample and left to cure for 24 h. Atmakuri et al. [82] employed the hand lay-up technique in the fabrication of Caryota and sisal natural fiber hybrid epoxy composites. Firstly, the epoxy resin and hardener were mixed in a ratio of 10:1. The mixture was stirred in a plastic bag for 3–4 min for proper mixing and thereafter left for 30 s. The epoxy resin mixture was poured over the fiber uniformly and compressed for a curing period of 24 h with a constant load of 5 kg to obtain a caryota and sisal fiber hybrid epoxy composite. The preparation of kenaf/hybrid boron nitride epoxy composites was reported by Xia et al. [83]. Sodium hydroxide-treated kenaf was mixed with hybrid boron nitride in deionized water and dried at 105 °C for 24 h. The preformed mat was completely mixed with epoxy. The mixture was then transferred to the steel mold and then compressed by a hot press at 150 °C and 13.2 MPa, and then maintained at this temperature for 60 min. The composite was removed and kept for 7 h for further use. A combination of the hand lay-up technique and the compression molding method has been used in the processing of a date palm/bamboo hybrid composite [84,85]. In their study, a mixture of fibers and epoxy resin together with hardener was stirred at room temperature with a stoichiometric ratio of epoxy to hardener of 2:1. Subsequently, the mixture was poured into a steel mold with a fiber-to-epoxy ratio of 50:50 wt% and transferred to a hot press heated to 110 °C for 10 min. It was then removed from the machine after it had cooled down to 10 min to obtain a date palm fiber/bamboo epoxy hybrid composite. The vacuum-assisted resin infusion molding technique [86] was employed in the fabrication of an epoxy composite containing 30 vol.% Boehmeria nivea fabric. Before composite preparation, Boehmeria nivea fabric was washed in deionized water for 20 min and dried in an oven at 80 °C for 24 h. A mechanical pump was used to vacuum the system at 760 mmHg. The composite sample was cured at room temperature for 24 h.

**Figure 2:**
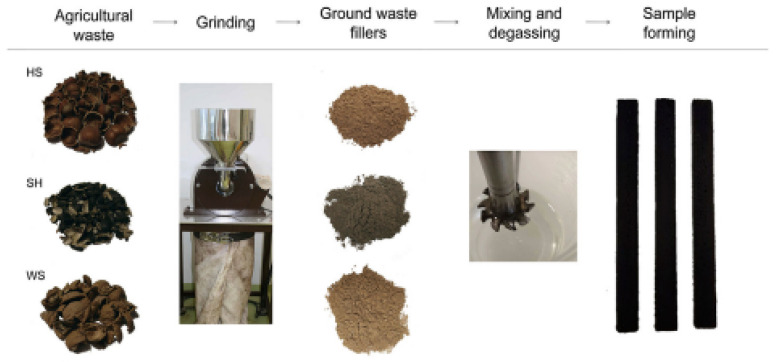
Preparation of epoxy-based natural fibers composites [87] (Reprinted/adapted with permission from Elsevier Ref.5457271400523 Copyright 2019).

The preparation of epoxy containing three different biofillers (walnut shell, hazelnut shell, and sunflower husk) is detailed in the report [87]. Composite constituents were mixed using a high-speed mechanical stirrer at three different rotational speeds (7000, 10,000, and 17,000 rpm) for 3, 1.5, and 0.5 min, respectively. Then the mixtures were degassed in a vacuum for 1 h and the curing agent was incorporated. Thereafter, the mixtures were stirred for 2 min at 7000 rpm and degassed for a period of 10 min. The samples were then cured for 2 days in Teflon form at room temperature and post-cured for 2 h at 80 °C. The preparation process is demonstrated in Figure 2 below.

**Figure 3:**
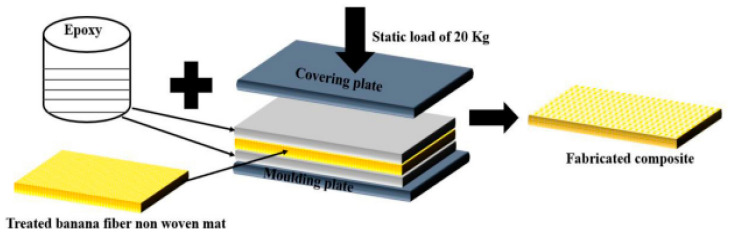
Preparation of woven banana epoxy-based composite [90] (Reprinted/adapted with permission from Elsevier Ref. 5457190666636 Copy right 2021).

The preparation of epoxy-containing seashells and glass fiber was reported by Krishna et al. [88]. In this synthesis, the epoxy, glass fiber, and seashells were mixed, and the hand lay-up process was employed in the processing of the composite. First, the glass fiber was laid followed by seashell powders. The layer composition in the metallic mold was compressed using a hydraulic cold press at 5 bars for 24 h. After 24 h, the composite was removed from the die and was ready to be characterized. The preparation of date palm fiber in phenolic epoxy composites was presented in another paper [21]. The fabrication of the composite was performed in a mold via hot pressing at 140 °C for 8 min. Thereafter, the mold was cooled and the composite was removed for characterization. Furthermore, the preparation of epoxy-containing synthetic fiber (Kevlar) and natural fiber (kenaf) was presented in another work [22]. First, the epoxy resin was mixed with the hardener in a ratio of 10:1 to promote the bonding of the epoxy matrix with the fibers. The Kevlar and kenaf fibers were stacked in alternate layers in the mold and then the epoxy resin was applied. After the application of epoxy resin, a roller was applied to promote better bonding. The composite was then pressed at 60 °C for a period of 8 h. The fabricated composite was then removed for mechanical characterization. Moreover, the fabrication of hybrid epoxy-based composite material using the hand lay-up technique was demonstrated in the work [89]. The epoxy-containing jute and carbon fibers were prepared in a split mold. The fibers were laid at the bottom of the mold, and the epoxy-containing hardener was brushed over it. The composite was kept in a hydraulic press at room temperature and a pressure of 0.5 MPa for 2 h. The base of the mold was removed from the hydraulic press and kept for 24 h. The fabricated composite was then removed and kept for 1 week for curing. Gairola et al. [90] fabricated a banana-fiber-reinforced epoxy-based composite using the hand lay-up technique. The epoxy resin was mixed with a hardener in a ratio of 10:1 and then the first layer was applied to the bottom of the mold and banana fiber was placed over it. Subsequently, the epoxy mixture was poured over it to impregnate the fiber thoroughly. A static load of 2 kg was placed over the upper plate for 48 h at room temperature to produce the composite, as shown in Figure 3. Biodegradable rice husk epoxy composites have also been prepared [11,12]. After the treatment of the rice husk with hot water and a sodium hydroxide solution, the epoxy-containing hardener was added to the rice husk one after the other, and consequently, single-layer, double-layer, and triple-layer bidirectional cross-fiber composites were produced. The prepared composites were left for 24 h to harden. Helaili et al. [8] fabricated an epoxy/alfa fiber biodegradable polymer composite. The epoxy resin and hardener in the ratio of 100: 26.4 were mixed using a magnetic stirrer for 30 min. After homogenization, the mixture was degassed in a vacuum to remove any residual gasses. The fiber was randomly spread in the mold and then the epoxy solution was cast into the mold. Subsequently, the remaining air bubbles were removed, and the sample was heated, cured for 2 h at 90 °C, and finally cooled to room temperature.

The fabrication of fiber composite materials with epoxy matrix involves two features: First, the fiber chemical structure, which affects the curing reaction, and this phenomenon occurs in restrained conditions. Secondly, because of the high-volume content of the filler fibers, the binder permeates the composite in a thin interlayer fashion. Generally, the curing of epoxy oligomers is characterized by a reduction in volume [91]. The curing of epoxy-fiber composites is dictated by the process parameters and this comprises time, temperature, and pressure [92]. Most importantly, the curing conditions and the nature of the component fillers are the factors that influence the kinetics of binder curing. Depending on the condition of the curing process, internal stresses arise due to shrinkage. Thus, contracting because of the curing reaction of the matrix should be compensated for through the flow of the binder from the outside layer to the inner layer, and this will prevent pore development during composite processing. It has been discovered that in the course of the curing reaction, the change in pore size is affected by the initial pressure, initial concentration of water, and diffusion coefficient [93].

In brief, the performance characteristics of epoxy natural fiber polymer composites rely on factors including component material properties, moisture, temperature, fiber volume fraction, orientation and straightness, and the presence of voids. To produce epoxy natural fiber composites of improved properties, the selection of the processing techniques and process parameters are very essential.

## 3. Mechanical Properties of Epoxy-Based Natural Fiber Biocomposite Materials

**Figure 4:**
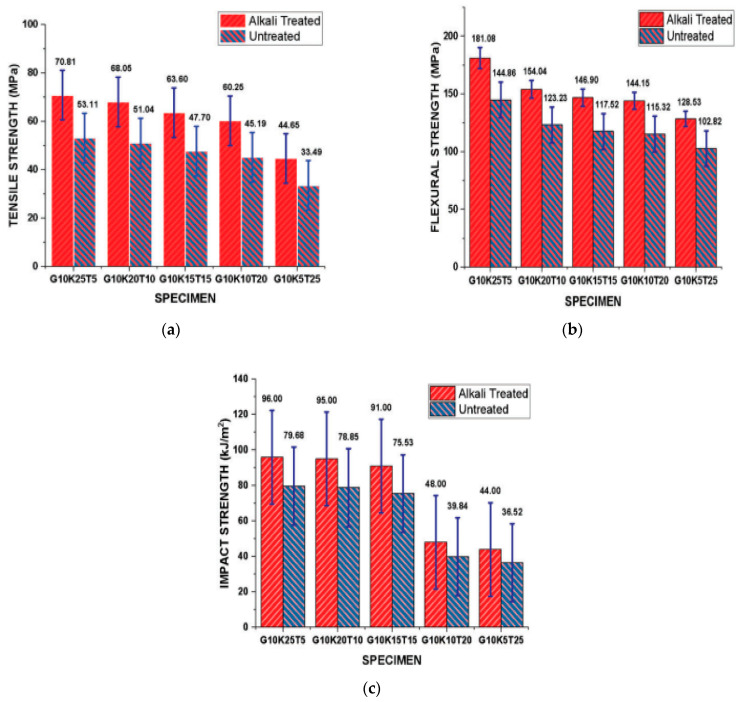
Mechanical properties: (**a**) Tensile strength, (**b**) flexural strength, and (**c**) impact strength of epoxy-based hybrid composites [66] (Reprinted/adapted with permission from Elsevier Ref. 5457191256269 copyright 2019).

The mechanical properties of natural fiber composites are generally lower than synthetic fiber composites. The natural fiber composites’ properties can be enhanced by incorporating them with synthetic fibers and applying preprocessing treatments on the natural fibers [20]. Table 1 lists the mechanical properties of natural fiber composites and natural fiberglass hybrid composites. Many studies of natural fiber composites and natural fiberglass hybrid composites focused on the mechanical properties of the composites and the effect of different factors, for example, the fiber content, treatments, fiber lengths, stacking sequences, and temperature on the properties. Increasing fiber content causes stress transformation from the epoxy matrix to the natural fiber in composites [94]. Prabhu et al. [66] studied the mechanical properties of epoxy-containing glass fiber, kenaf fiber, and waste tea leaf hybrid composites. The glass fiber was kept at 10 wt% while the weight fraction of kenaf and waste tea leaf fibers varied from 5 to 25 wt%. Maximum mechanical properties (tensile, flexural, and impact strength) were achieved at 10 wt% glass fiber, 25 wt% kenaf fiber, and 5 wt% waste tea leaf fiber (Figure 4). The mechanical properties were found to decrease with an increasing concentration of waste tea leaf fiber but increase with an increasing weight fraction of the kenaf fiber in the hybrid composites. However, a higher fiber content can cause insufficient epoxy matrix and fiber wettability and, as such, more voids within the matrix, which in turn cause weak adhesion as the void serves as a stress concentrator. Alkali treatment, which is a common treatment used for composites, decreases moisture absorption, separates the fibers, cleans the surface, and increases cellulose crystallinity at lower concentrations in contrast with damaging the fiber surface at higher concentrations. Decreasing the natural fiber content at the expense of increasing the glass fiber content increases the tensile, flexural, impact, and hardness properties of the hybrid composite, and this is because glass fibers have higher mechanical properties than the used natural fibers.

### 3.1. Tensile Properties

Two main tensile properties, namely, strength and stiffness, have been investigated by researchers in natural fiber composites. Studies have shown that the tensile properties of the composites increased when increasing the fiber content [105,109,113,119]. Other studies revealed that this is true up to an optimum value in another type of fiber composites [106,116,117]. The alkali treatment concentration was reported to increase to an optimum value, and after that, decreased the tensile properties [101,109,115,117]. This is because the high concentration of NaOH could destroy the natural fibers, thereby reducing the reinforcing potency. Aligned-fiber and long-fiber composites have better tensile properties than random-fiber and short-fiber composites, respectively [103,109]. Increasing the temperature has been shown to decrease the tensile properties of fiber composites [115].

In natural fiberglass hybrid epoxy composites, increasing the fiber content with a fixed glass fiber volume fraction has a similar effect as the addition of fibers in natural fiber composites [98]. Studies have reported that decreasing natural fibers and increasing the glass volume fractions increases the composite tensile modulus and increases its tensile strength to an optimum value before decreasing [112,118]. This is because the void content of the composite is reduced with increasing fiber content up to the optimum value. Beyond the optimum value, bonding the fiber with the matrix becomes difficult, likely due to agglomerations, and that results in a weak interface between the fiber and the epoxy matrix leading to poor mechanical properties [123]. The fiber’s orientation and layer-stacking sequence can affect the tensile properties, for example, natural fibers and glass layers bound to one another have more tensile properties than changing layers stacking sequences, and a hybrid composite with fibers aligned along the load has the best tensile properties [100,102,108]. The glass bonding fiber layers were shown to have higher tensile properties than fiber bonding glass layers [114].

### 3.2. Flexural Properties

The flexural strength and modulus were also investigated in natural fibers composites. Increasing the fiber volume fractions in the composite increases its flexural properties [105,113,119,121]. Increasing the alkali treatment concentration was shown to increase the flexural properties to an optimum value and then thereafter decreased them [101,117], and this is likely due to the destruction of the natural fibers by the high concentration of the NaOH solution used in the treatment. Aligned-fiber and long-fiber composites are shown to have better flexural properties than random-fiber and short-fiber composites, respectively [103,109]. Changing natural and glass fibers volume fractions have effects on the flexural properties, as discussed on the tensile properties [98,112,118]. Generally, the natural fibers bonded by glass layers have the highest flexural properties among other sequence layers, and it is reported that switching sequence layers have higher flexural properties and aligned natural fibers showed higher flexural properties than other orientations [100,102,108,114].

### 3.3. Impact Properties

Increasing the fiber volume fraction in the composite was reported to increase or decrease the impact energy [106,113,117,119]. Increasing alkali treatment concentration was shown to increase the impact strength up to an optimum level, and afterward, it decreases [101,109]. Again, this decrease in impact energy with increasing concentrations of the alkali solution is attributed to the destruction of the natural fibers by the alkali solution at high concentrations. The impact energy increases when increasing the fiber length [103,109]. The gradual exchange of natural fibers with glass fibers increases the impact strength to an optimum value, then it decreases beyond the optimum value [118,122] due to a reduction in the bonding ability of the fibers with the epoxy matrix leading to high void contents and weak interfacial adhesion between the epoxy and reinforcing fibers.

### 3.4. Hardness Properties

Hardness decreased as the natural fiber content increased at the expense of the epoxy matrix in glass hybrid composites [98]. Natural fiber layers bounding glass layers have higher hardness than interchanging layer sequences [100]. Longer natural fibers in a glass hybrid composite were shown to have higher hardness than composites with shorter fibers [103,104]. It is important to state that the hardness of natural fiber/epoxy composites strongly depends on the level of distribution of the natural fiber in an epoxy resin matrix. A better, more uniform distribution of the natural fiber in an epoxy matrix enhances the mechanical hardness of the composites.

In summary, the mechanical properties of epoxy/natural fiber composites vary with the volume concentration of the fiber in the epoxy matrix. Depending on the natural fiber type, maximum mechanical properties are usually achieved at fiber concentrations ranging from 20–48 wt% [119,124,125,126]. Beyond the optimal value, the properties are reduced due to weak bonding between the fiber and the matrix. Furthermore, the mechanical properties of the composites are equally sensitive to moisture absorption, and the exposition of epoxy/natural fiber composites to moisture reduced their mechanical properties and dimensional stability [127]. In addition, mechanical properties equally increased with the fiber surface modification before their inclusion in the epoxy matrix. Treated natural fiber/epoxy composites have shown superior mechanical properties as compared to the untreated natural fiber/epoxy composites counterparts [101,128,129,130]. This is because fiber treatment results in the partial dissolution of lignin and amorphous cellulose and the consequent splitting of the fibers into smaller sizes. This facilitates permeating into gaps of the fibers for epoxy resin and forming effective interfacial adhesion [128].

## 4. Physical Properties of Epoxy-Based Natural Fiber Polymer Composite Materials

Water absorption and density are two commonly evaluated major physical properties of epoxy/natural fiber composites.

### 4.1. Water Absorption

The moisture absorption characteristics of natural fibers are one of the considerations in the choice of natural fibers as reinforcement in the epoxy matrix. This is because dimensional stability, mechanical tensile strength, electrical properties, swelling, and void formation are all affected by moisture content. Cellulosic fibers are highly hydrophilic in nature due to the presence of hydroxyl groups and hydrogen bonding and their capability to cause hydrogen bonding with water and moisture [131,132]. Generally, as the cellulose content of the natural fiber increases, the water absorption and thickness swelling decrease. The percentage of water absorption, $W_{At}$, characteristics of epoxy/natural fiber composites after a certain period of time are measured using Equation (1),

(1) WAt=Wi−WtWt×100

where $W_{t}$ denotes the weight of the sample before immersion in water and $W_{i}$ represents the wet weight of the sample after it has been immersed in water for a certain period.

The water absorption properties of cassava starch hybrid epoxy and sugar palm fiber epoxy composites were studied by Edhirej et al. [133]. After 1 month of immersion, the moisture content of a sugar palm fiber epoxy composite was 6.01% while that of cassava bagasse epoxy composites was 14.92%. This implies that cassava bagasse is more hydrophilic than sugar palm fiber.

Chen et al. [45] studied the water absorption behavior of untreated and silane-treated glass fiber in epoxy composites and discovered that the water uptake of the composites increases with an increase in the weight fraction of the glass fiber for both treated and untreated samples. Moreover, the rate of water absorption of the composites was high initially and gradually decreased until the saturation level was attained. It was conceived that the water absorption behavior of the composites could be due to extra spaces at the glass fiber and epoxy interface, which permits the sorption of water molecules. Baig and Mushtaq [134] investigated the water absorption behavior of an epoxy composite containing 50 wt% and 70 wt% tamarind shell fiber fabricated by the hand lay-up technique. The percentage of water absorption of epoxy-containing 50 wt% and 70 wt% tamarind fiber after 48 h of immersion was 2.91 and 4.03%, respectively. It was evident that the water absorption behavior of the composite increased with the concentration of tamarind fiber in the epoxy matrix. The composite containing 70 wt% tamarind showed a greater rate of water absorption than those containing 50 wt% tamarind fiber. As explained by the authors, tamarind fibers contained hydroxyl groups that promoted water absorption characteristics of the composites. Ranakoti et al. [135] compared the rate of water uptake of untreated and alkaline-treated tasar silk fiber in an epoxy matrix. The amount of tasar silk fiber for both treated and untreated samples varied from 5 wt% to 30 wt%. The water absorption rate increased with an increase in the content of tasar silk fiber. However, the water absorption rate was higher in untreated tasar silk fiber epoxy composites than in treated tasar silk epoxy composites counterparts. This is because alkali treatment of fiber reduces the hydrophilic character of the fiber leading to the reduction or elimination of the hydroxyl groups from the surface of the fiber and thereby minimizing the water uptake. Moreover, treated tasar silk fiber epoxy composites exhibited better interfacial bonding between the fiber and the matrix with a subsequent reduction in the void present in the composites. This equally contributed to the lower water absorption rate of the treated samples as the voids serve as storage media for water in the composites.

Generally, the rate of water absorption of epoxy-containing natural fibers composites depends on the volume content of the fiber in the epoxy, the nature of the fiber, surface modification of the fiber, and the number of voids present in the composites [40,136,137,138]. An increase in the concentration of natural fibers in the epoxy matrix promotes water absorption characteristics of the composites. Furthermore, treated fibers in the epoxy matrix composites have a lower rate of water absorption than the untreated natural fiber epoxy composites.

### 4.2. Density

The density of composites can be measured using a densimeter based on Archimedes’ law by ASTM D2734. The density of epoxy natural fiber composites depends on the type of fiber, the volume content, the surface treatment, and the strength of interfacial adhesion between the natural fiber and epoxy [139]. The theoretical density of the composite materials in terms of the weight concentration of the different constituents can be evaluated using Equation (2):

(2) ρct=1Wfρf+Wmρm

where $\rho_{ct}$ is the density of the composites, $W_{f}$ is the weight fraction of fiber, $\rho_{f}$ is the density of fiber, $W_{m}$ is the weight fraction of the matrix, and $\rho_{m}$ is the density of the matrix [104].

Thus, the void content of the composites can be determined as per the ASTM D-2734-70 standard procedure. The void volume fraction, $V_{v}$, of the composite is estimated using Equation (3):

(3) $$V_{v}=\frac{\rho_{ct-}\rho_{ce}}{\rho_{ct}}$$

where $\rho_{ct}$ is the theoretical density and $\rho_{ce}$ is the experimental density of the composites.

Sulardjaka et al. [139] studied the density and porosity of epoxy-containing woven water hyacinth fiber composite material with the weight fraction of the fiber range from 0–35 wt.%. The density of the composite decreased with an increasing weight fraction of the woven water hyacinth fiber. This is because as the concentration of the fiber increased, the weight fraction of the epoxy was reduced, and this results in a density reduction of the composites. The porosity of the composites was increased with an increasing weight fraction of the fiber in the epoxy matrix. For example, the addition of 35 wt% of woven water hyacinth fiber to the epoxy matrix increased the porosity from 0.35% to 13.82%. The entrapment of the air due to inhomogeneity in the fiber structure is responsible for the increase in porosity. In another report, Ranakoti et al. [135] explained that the void fraction of epoxy/natural fiber composites increased with an increasing concentration of the fiber in the epoxy. However, untreated fiber epoxy composites have a higher void fraction than treated fiber epoxy composites. They equally explained that experimental density is lower than theoretical density because of the presence of voids in composites during the manufacturing and curing process. Thus, the number of voids in the composites could be reduced through better distribution of the natural fiber in the epoxy matrix.

## 5. Electrical Properties of Epoxy-Based Natural Fiber Polymer Biocomposite Materials

Natural fibers have been acknowledged as a replacement for synthetic fibers in the development of electrical and electronic devices [140]. The electrical properties of epoxy natural fiber biocomposite materials relevant for this purpose include the dielectric constant (έ), loss factor (ἒ), dissipation factor (tan *δ*), and electrical conductivity.

### 5.1. Dielectric Properties of Epoxy-Based Natural Fiber Biocomposites

The dielectric constant (έ) of a material is defined as the ratio of the capacitance of the condenser containing material to a similar condenser under vacuum. The dielectric constant of materials mainly depends on temperature and frequency. The interfacial orientation and atomic and electronic polarization are the specific factors affecting the dielectric constant of composite materials. Interfacial polarization arises because of the differences in conductivities and polarization between the natural fiber and the polymer matrix. The dielectric constant of epoxy-based natural fiber composite materials can be estimated using Equation (4) [141]:

(4) έ=CC0

where *C* is the capacitance with a dielectric material and $C_{0}$ is the capacitance without the dielectric.

The $C_{0}$ in (pF) in this case can be determined using the relation:

(5) $$C_{0}=[\left(0.08854\;A\right)/d$$

where *A* (cm^2^) is the area of the electrode and *d* (cm) is the thickness of the sample.

Interestingly, both orientation polarization and interfacial polarization are affected by the concentration of the filler in the matrix. Generally, dielectric polarization increases with an increase in temperature but decreases with increasing frequency. Furthermore, the dielectric constant of epoxy-based natural fiber composites increases with fiber loading because of the gain in orientation and interfacial polarization as a consequence of the availability of polar groups in the natural fibers [38].

Loss factor (ἒ) is a term used to describe losses in the transmission and distribution of power over a given length of time. Similar to the dielectric constant, the loss factor varies with temperature and frequency. The loss factor is high at low frequencies and equally increases with increasing fiber loading.

The dissipation factor (tan *δ*) is the measure of the electrical energy that is converted into heat in an insulating material. The heat deposited in the insulating material increases its temperature and promotes the deterioration of the material. The tan *δ* can be evaluated using Equation (6).

(6) tanδ=ἒέ

Generally, the loss factor decreases with increasing frequency while it equally increases with fiber loading. This is because the increase in fiber loading results in an increment in the number of polar groups and facilitates orientation polarization [38,142,143].

**Figure 5:**
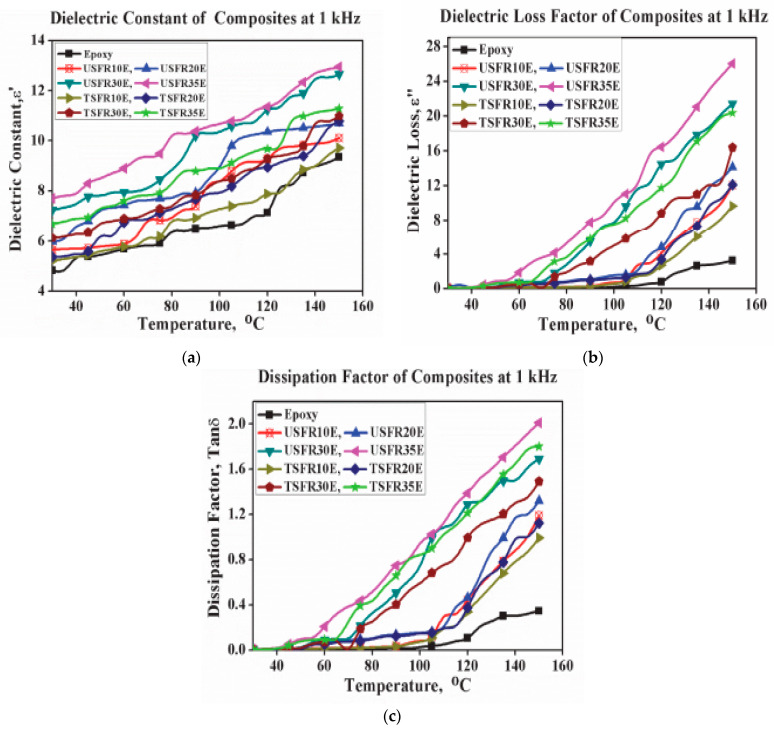
Electrical properties: (**a**) Dielectric constant, (**b**) dielectric loss, and (**c**) dissipation factor of untreated and treated sisal fiber-reinforced epoxy composites [142] (Reprinted/adapted with permission from IEEE Ref. 18162007 Copyright 2018).

Jayamani et al. [144] studied the dielectric constant of hybrid epoxy-based composite materials and the dielectric constant was found to increase with increased fiber loading, which was attributed to an increase in the number of polar groups due to lignocellulosic fiber inclusion. Furthermore, the dielectric constant of the hybrid composite containing alkali treatment fiber is lower than the untreated fiber composite of the same composition [145]. This might be because the alkali treatment reduces the hydrophilic character of the fiber and, subsequently, reduces the number of polar groups, hence the lower value of the dielectric constant of the alkali-treated epoxy-based composites. It was equally reported by Kumar and Kumar [36] after they studied the dielectric constant of silane-treated bamboo-epoxy nanocomposites and discovered that the dielectric constant of epoxy/natural fiber composite decreases with the natural fiber treatment. Omri et al. [37] explained that dielectric spectroscopy is capable of providing valuable information on both thermal and frequency characteristics properties of polymer composites. According to them, dielectric constant measurement of laminated composite materials could help in elucidating the adhesion of the reinforcement fillers in the epoxy matrix. Chand and Jain [141] observed that the dielectric constant of sisal fiber-reinforced epoxy matrix composites increase progressively with increasing fiber content while it decreases with the increase in fiber length and frequency. This is equally in agreement with another study [146]. Nimanpure et al. [142] reported dielectric constant values of 5.65 and 5.21 for 10 wt.% untreated and treated sisal fiber, respectively, in an epoxy matrix measured at a frequency of 1 kHz and a temperature of 30 °C. This value increased to 12.95 and 11.29 as the weight fraction of the fiber increased to 35 wt.%. The dielectric constant equally increases with increasing temperature (Figure 5a). In the same fashion, the loss factor and dissipation factor (Figure 5a,b) exhibited almost the same trend as they both increased with fiber loading and temperature as in the case of the dielectric constant. Sreekumar et al. [38] showed that the dielectric constant of polymer natural fiber composites is sensitive to frequency, temperature, the volume concentration of the fiber, and the fiber orientation. The sensitivity of the dielectric constant with a change in frequency is attributed to orientation and the interfacial polarization phenomena. The dielectric constant is low at a high frequency and high at a low frequency. This is because the complete orientation of the molecule is possible only at lower frequencies, and orientation polarization requires enough time to attain an equilibrium static field, unlike electronic and atomic polarization. As explained in a previous study [147], the dielectric constant, loss factor, and dissipation factor increase with the increase in temperature, and the reason for this is that, at high temperatures, the mobility of the polymer chains and interface polarization is enhanced.

Generally, the dielectric constant of epoxy-based natural fiber composite materials increases with fiber loadings and temperature but decreases with the frequency and fiber treatment. In addition, the dielectric constant of epoxy/glass fiber composites is lower than epoxy/natural fiber composites because of the absence of hydroxyl groups in the glass fiber [148].

### 5.2. Electrical Conductivity of Epoxy Natural Fiber Biocomposites

The electrical conductivity of natural fiber-reinforced plastics relies on the frequency, fiber content, and fiber length. The electrical conductivity increases with the fiber content and frequency [141]. The volume resistivity, which is the inverse of the electrical conductivity of polymer composites, decreases with fiber loading. It is of the utmost importance to realize that volume resistivity decreases with frequency. This reduction is attributed to the interfacial polarization occurring because of the inhomogeneity of the composite system. It is a well-known fact that in polymers, the majority of current flows through the crystalline part, and the passage of current through the amorphous part occurs only in the vicinity of moisture. Thus, the presence of hydroxyl groups in natural fibers aids in moisture absorption and enhances the conductivity of the matrix [38].

The volume resistivity (*ρ*) can be calculated using Equation (7):

(7) $$\rho =\frac{RA}{t}$$

where *R* is the resistance, *A* is the area of the cross-section of the sample, and *t* is the thickness of the sample. The electrical conductivity can then be evaluated using Equation (8):

(8) σ=1ρ

**Figure 6:**
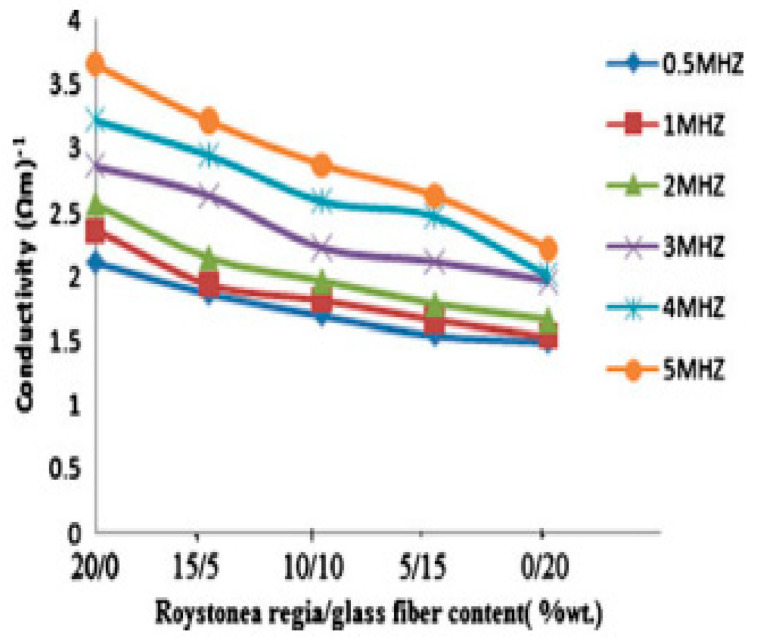
The electrical conductivity of hybrid fiber epoxy composites [148] (Reprinted/adapted with permission from Elsevier Ref.5457271065453 Copyright 2012).

Goud and Rao [148] studied the electrical performance of Roystonea regia/glass fiber epoxy composites with varying weight percentages of glass fiber in the composites (Figure 6). The electrical conductivity decreases with an increasing weight fraction of the glass fiber content while it increases with an increasing frequency. This is because the electrical conductivity of glass fiber is lower than the conductivity of Roystonea regia fiber. The hydrophilic nature of natural fibers has made them more conductive than synthetic fibers.

In general, the electrical conductivity of natural fiber epoxy composites increases with an increase in frequency and fiber loading. The electrical conductivity of treated natural fiber epoxy composites exhibits a lower value than the untreated natural fiber composites.

## 6. Thermal Properties of Epoxy-Based Natural Fiber Biocomposites

**Figure 7:**
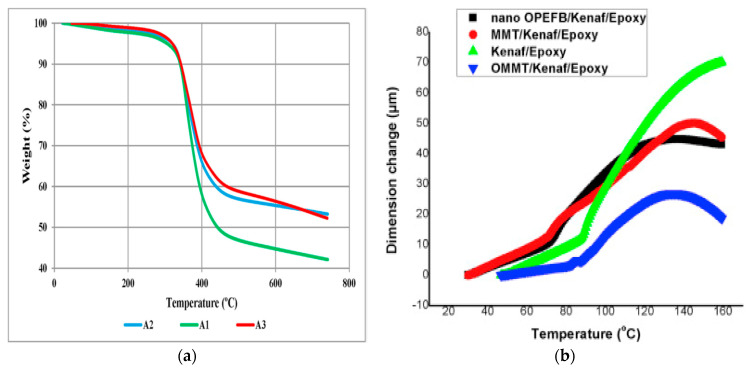

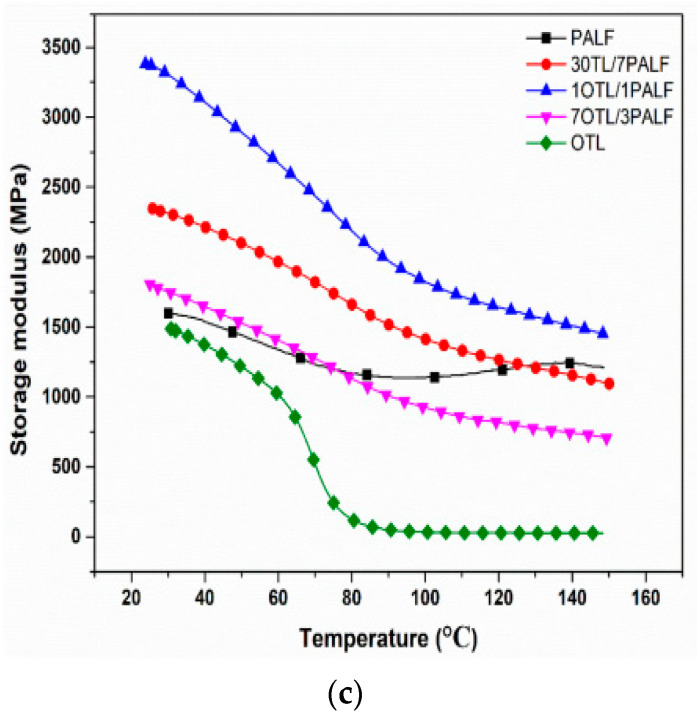
(**a**) TGA analysis thermogram for epoxy-CF modified coir-fiber at 30% (A1), 20% (A2), and 10% (A3), (**b**) TMA graph of kenaf/epoxy composites and filler-filled kenaf/epoxy hybrid nanocomposites, and (**c**) DMA graph of olive tree leaves/pineapple leaf hybrid epoxy composites [152,154] (Reprinted/adapted with permission from Elsevier ref.5457220515829 Copyright 2022).

A thermal analysis is conducted to determine the physical property of a substance as a function of temperature whilst the substance is subjected to a controlled temperature program. A range of techniques such as thermogravimetry, differential scanning calorimetry, thermomechanical analysis (TMA), etc., can be employed to investigate different thermal properties under temperature control. The thermal stability of the composite via thermogravimetry analysis (TGA) under a specific gaseous environment is beneficial to studying the temperature at which the degradation or decomposition of the composite would occur for practical purposes. In addition, the investigation of the thermal expansion, heat contraction, and absorption rate of moisture can be deduced through a thermal stability test. The TGA analysis is usually conducted with a sample heated to a higher temperature (between 600 and 1000 °C) at a constant heating rate while monitoring the degradation of material as a function of weight loss. The thermal stability, as well as thermal conductivity and diffusivity, of a fiber-epoxy composite is enhanced with the incorporation of fiber in an epoxy polymer matrix [149]. As reported by Yadvinder Singh et al. [150], there was an increase in the thermal stability of the coir fiber-epoxy composite from 10% to 30% loading, accompanied by lower weight loss in the composite samples as a function of higher fiber loading (see Figure 7a) [150]. Differential scanning calorimetry (DSC), under the thermal analysis technique, is useful for providing the glass transition (T_g_) and variation of T_gs_ due to the composite composition. TMA is a thermal analysis technique used to evaluate the dimensional changes (expansion or contraction) of a composite, under mechanical load or stress, as a function of temperature in X, Y, and Z directions [151]. The dimensional stability and mechanical compatibility of composites are connected or expressed in terms of the thermal expansion coefficient (CTE) from the broader perspective of composite engineering. The observation of the TMA graph provides information about the rubbery or glassy state of the composite, and the incorporation of nanofillers (such as OPEFB, MMT, and OMMT) on the epoxy-kenaf matrix improved the CTE significantly as shown and reported in Figure 7b by [152]. Visco-elastic properties of epoxy-based natural fiber composites are usually studied using dynamic mechanical analysis (DMA). DMA compares the storage modulus of the materials concerning a range of temperatures [153]. The test is performed using a DMA analyzer in three-point bending mode at a given heating rate and range of temperatures. SenthilKumar et al. [154] studied the visco-elastic properties of olive leaves/pineapple leaf fibers epoxy hybrid composites using DMA at a temperature ranging from 30 to 150 °C. The loss modulus of the hybrid composites was found to be higher than that of pure epoxy (Figure 7c). As the storage modulus represents the stiffness or elastic modulus of the material, the higher values achieved for hybrid composites showed that the hybrid composites have a superior storage modulus, which was a result of the uniform dispersion of the fillers in the epoxy matrix. Generally, the storage modulus decreases with increasing temperature, which usually indicates a phase transition from a glassy state to a rubbery state. Higher Tg connotes a delay in the phase transition from a glassy to a rubbery state, and this represents superior thermal stability for the hybrid composites [155].

**Figure 8:**
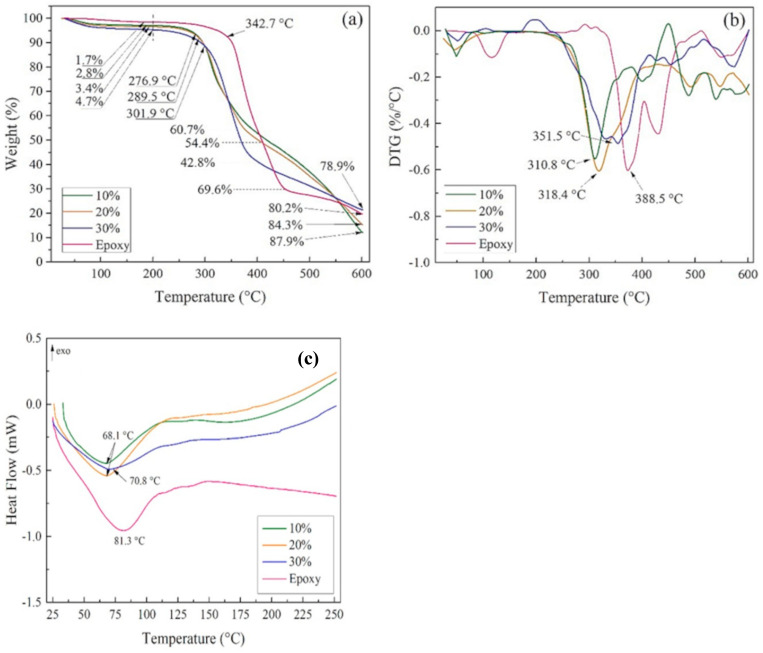
TGA (**a**), DTG (**b**), and DSC (**c**) curves of epoxy resin and epoxy/hemp fiber composites [159] (Reprinted/adapted with permission from Elsevier Ref.5457220890548 Copyright 2021).

Knowledge of the thermal properties of composites is of great importance when the need to meet new requirements of polymer matrix composite designs arises. Different new materials with unique properties that meet the needs of a particular purpose are currently under research in different parts of the world [156,157]. Agrawal et al. [158] developed a mathematical model for determining the effective thermal conductivity of hybrid-filled composites. In [158], aluminum nitride and aluminum oxide were used as conductive fillers, while pine wood dust and rice husk were used as insulation fillers. The mechanical, morphological, and thermal properties of mixed glass fiber and cellulose nano-fibers (CNFs) were investigated by Taufik Azhary et al. [7]. Under a nitrogen atmosphere, a 30–800 °C temperature range, and a heating rate of 10 °C/min, they carried out a thermo-gravimetric analysis (TGA) using the TG/DTA Hitachi STA7300 machine. From the TGA and derivative thermo-gravimetry (DTG) curves of the composite mixture with 1 wt% CNF, which indicates a three-step degradation process, it was concluded that the addition of CNFs did not affect the thermal resistance of the epoxy/glass fiber composite. Recently, Ribeiro et al. [159] investigated the thermal and ballistic performance of epoxy reinforced with cannabis sativa hemp fabric using differential scanning calorimetry and thermogravimetric analysis. The volume fraction of the fiber varied from 10–30% and the TG/DTG analysis was conducted at a heating rate of 10 °C/min in a range of 30–600 °C, while the DSC was conducted from 20–250 °C at a heating rate of 10 °C /min. As shown in Figure 8a,b, the thermal behavior of the composite seems to be influenced by the volume fraction of the hemp fiber. The composites present an initial peak up to 100 °C and this represents moisture desorption. The neat epoxy resin has a lower mass loss (1.7%) as compared to the composite with a mass loss (2.8–4.7%). This disparity in the mass between the epoxy and composites is because of the low water content of the epoxy due to its hydrophobic nature. Furthermore, epoxy/hemp fiber composites have their onset temperature (T_onset_) earlier, along with the maximum temperature (T_max_) as compared to epoxy resin. The reason for this is that the lignin component of the fiber starts degrading at approximately 200 °C [160]. For the composite, the onset temperature (T_onset_) ranges from 276.9 to 301.9 °C and T_max_ from 310.8 °C to 351.5 °C. For the neat epoxy resin, the T_onset_ begins at approximately 342.7 °C and its T_max_ was at 371.4 °C meaning that the composites have lower thermal characteristics than the neat epoxy resin [160,161]. Figure 8c shows the DSC curves of the neat epoxy resin and the composites reinforced with hemp fabric fiber. For the neat epoxy, an endothermic peak was observed at 81.3 °C, and this is related to the glass transition temperature (T_g_) [162]. The inclusion of hemp fiber of varying volume fractions into the epoxy resin causes the displacement of the endothermic peak ranging between 68.1 and 70.8 °C. This change is attributed to the release of moisture present in the hydrophilic hemp fiber and the T_g_ of epoxy resin, which both contributed to the increasing T_g_ of the composites.

In other research, a study on the enhancement of the thermal behavior of fiber-reinforced epoxy hybrid composites of a date palm/bamboo mixture was carried out [10]. The significance of hybridization was demonstrated by comparing the date palm fiber (DPF)/bamboo fiber (BF) hybrid composite to bamboo-fiber-reinforced epoxy by investigating their dynamic-mechanical, thermal-mechanical, and thermal properties. With the use of a nitrogen atmosphere at a heating rate of 20 °C/min under a 30 to 700 °C temperature range, the thermal stability of all composite samples was tested by TGA using TGA Q500 V20.13 Build 39. In addition to TGA, thermo-mechanical analysis (TMA) was carried out using a TMA Q400 V22.5 Build 31 setup at a heating rate of 5 °C/min under a temperature range of 30 to 200 °C while using a nitrogen atmosphere. With the addition of date palm fiber in epoxy resins, there was a slight improvement in the thermal decomposition of all composites, thereby increasing the overall thermal stability of the hybrid composites.

In summary, the addition of natural fibers to an epoxy matrix is expected to lower the thermal stability of the resulting composites [9,34,35,159,160,162] due to the hydrophilicity nature of the natural fibers. However, the hybridization of natural fibers with synthetic fibers such as glass fibers is expected to improve the thermal stability of the composites [163].

## 7. Characterization of Epoxy-Based Natural Fiber Polymer Composites

Understanding the characteristics and performance of the epoxy-based natural fiber polymer composite requires physico-chemical investigation through different characterization techniques viz. SEM, XPS, XRD, FTIR, TGA, NMR, and so on. The elucidation of these characteristics’ information provided a scientific guide and information related to the performance, modification, and optimum use of the composite for societal and industrial purposes. The aforementioned techniques provide necessary information about the stiffness, fatigue performance, strength, thermal expansion, glass transition temperature, corrosion resistance, absorption rate, and energy consumption for scientists and engineers in deciding the effective industrial use of the composite.

### 7.1. Microscopic Techniques

**Figure 9:**
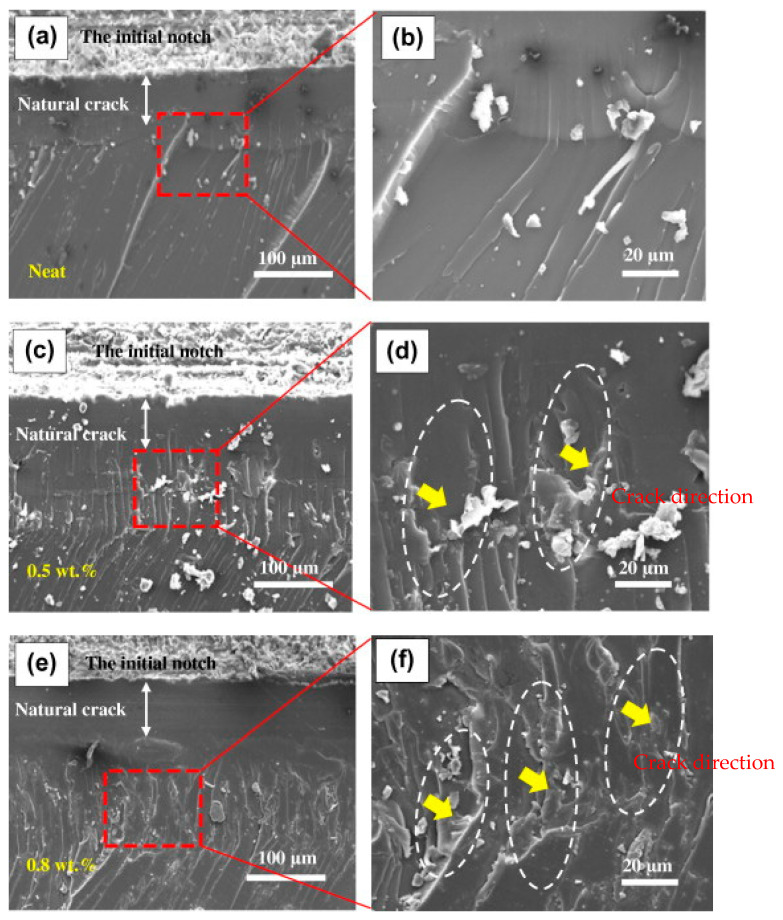
SEM analysis of fractured surfaces of pure epoxy: (**a**,**b**), 0.5 wt.% MBF (**c**,**d**) and 0.8 wt.% MBF (**e**,**f**) with the direction of crack propagation [168] (Reprinted/adapted with permission from Elsevier Ref.5457230729040 Copyright 2013).

Microscopy techniques such as scanning (SEM), transmission (TEM), and atomic force (AFM) microscopy are commonly employed to characterize the modified fiber and/or fiber-epoxy matrix to understand the interfacial interaction, wettability, and absorption rate [123,164]. SEM analysis of an epoxy composite modified with natural fiber provides information about the texture, morphology, and topography. The SEM analysis can be used to examine the surface roughness, texture, size and shape, bonding, and interfacial adhesion of the epoxy-based polymer composite modified with natural fiber, and is ultimately used to project the mechanical and other properties of the composite [165]. Yadvinder et al. [150] revealed that SEM analysis could be used to examine the bonding between epoxy components and hydroxyl ions. Furthermore, the treatment of coir fiber with alkali (NaOH) in an epoxy composite exhibited important structural and textural morphology, where the SEM micrograph revealed a smooth texture, indicating improved intermolecular bonding of the composite [166] compared to the non-treated one. The fracture analysis (fractography) of epoxy-fiber composite can be observed through SEM micrographs when mechanical testing (flexural or tensile) is conducted. SEM monitors the failure of the composite with fiber pulling out, and the extent of interfacial bonding in the fiber-epoxy matrix can be revealed for practical purposes [167]. An important study of carbon fiber-epoxy resin (CF/EP) modified with micro/nano bamboo fibrils (MBFs) was conducted to investigate the influence of composites on mechanical properties [168]. As shown in Figure 9, the fractured analysis of the pure epoxy and modification with MBFs (at 0.5–0.80 wt.%) was examined. The micrograph analysis revealed the toughening mechanism of epoxy-resin modified with MBF, where the pure epoxy (Figure 9a,b) exhibited a smooth and glassy surface with micro-flow lines. Conversely, the MBF@ 0.5 wt.% in epoxy (Figure 9c,d) had a fractured surface characterized by a shear-like and stick-slip pattern, and an increase in MBF to 0.8 wt.% (Figure 9e,f) produced a rough, fractured surface characterized by a jagged multi-plane pattern. Furthermore, the SEM analysis via micrograph observation provides an indication of the crack direction, fatigue strength, etc., as detailed in the study by [168]. Energy dispersive spectroscopy (EDS or EDX) complements SEM techniques to obtain the elemental compositions in the matrix composite, though a well-established technique such as XPS or XRF can be availed for more accuracy.

**Figure 10:**
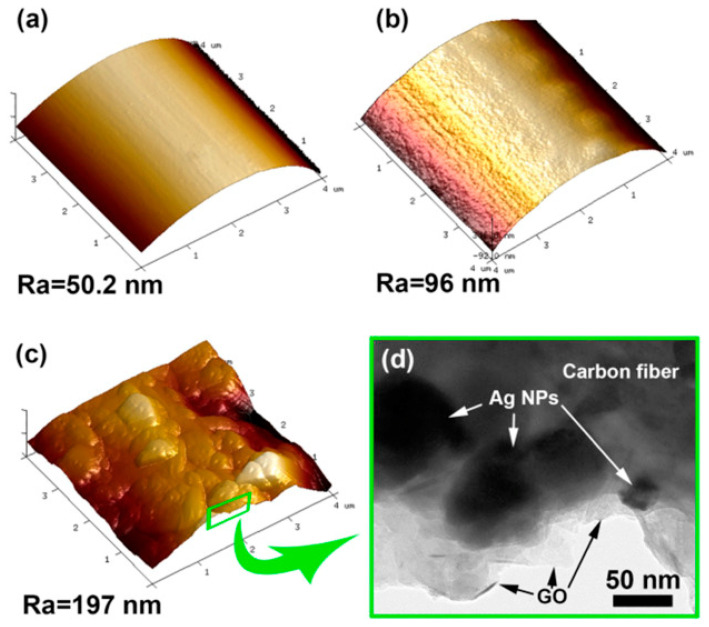
AFM images of (**a**) untreated CF, (**b**) CF/Ag-30, (**c**) and CF/Ag/GO-60 and (**d**) TEM image of cross-section of CF/Ag/GO-60 [170] (Reprinted/adapted with permission from Elsevier Ref. 5457230991168 Copyright 2014).

Atomic force microscopy (AFM) is a characterization technique utilized to probe the morphology and topography of the epoxy-fiber composite and observe the mechanical locking. Wang et al. [169] investigated the incorporation of silver nanoparticles (Ag-NPs) and graphene-oxide (GO) on a carbon fiber (CF)-modified epoxy-resin to improve the tensile strength (by filling the surface cracks of fibers) and mechanical interlocking. The surface roughness for CF and CF/Ag-GO at different electrodeposition time intervals (10–90 s) was observed by AFM (Figure 10a–c). An increase in the surface roughness was observed from untreated CF and treated ones, and the value reached the highest for CF/Ag/GO-60 at Ra = 197 nm, representing good mechanical interlocking between CF and the epoxy. The interfacial strength of the composite was examined by transmission electron microscopy (TEM), where the microstructural observation provides information about the structural incorporation of modifiers in the fiber or epoxy matrix. In the same study by [169], the successful incorporation of Ag and GO into the CF matrix was observed, and GO was seen as a sheet and Ag as spherical NPs below 100 nm as shown in Figure 10d. This observation confirmed the formation of a new hierarchical structure exhibiting the interfacial strength needed for practical applications [170].

### 7.2. Fourier Transform Infrared (FTIR)

**Figure 11:**
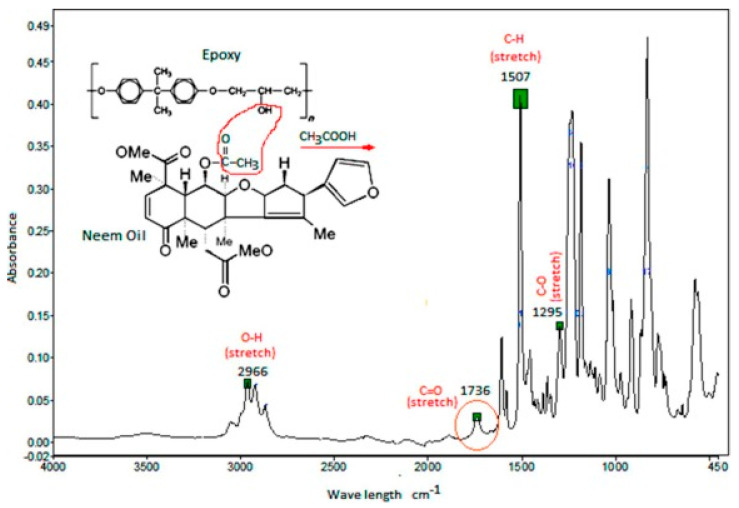
FT-IR spectra of acetic acid in epoxy-neem matrix [174] (Reprinted/adapted with permission from Elsevier Ref. 5457231434763 Copyright 2019).

The investigation of functional groups in the fiber-epoxy matrix is relevant to the interfacial bonding that exists before and after composite formation and is important to other characteristic information (mechanical (tensile and flexural) properties, water absorption, and other properties). The Fourier Transform Infrared analysis provides technical information about functional groups and chemical composition in the fiber-based epoxy. For example, the study by Sumesh et al. [171] where banana (BFA), pineapple (PFA), and coir (CFA) fly ash were studied in the epoxy composite revealed different functional groups associated with each precursor of fiber. The O-H stretching characteristic band in the composite matrix represents the presence of water absorbed, phenol, and alcohol usually found at approximately 3500 cm^−1^. The octahedral form of aluminum (Al(OH)_6_) in the fly ash fibers indicating symmetric and asymmetric vibrations is found between 770 and 810 cm^−1^, while the Si-O-Si asymmetric vibration band is observed at 1138 cm^−1^. The C-H stretching was found at approximately 2915 cm^−1^ for BFA and CFA and 2970 cm^−1^ for PFA. The characteristic band of lignocellulosic fiber composites is usually observed at 3360–3400 cm^−1^ [172,173]. The absorbed water epoxy bending bands in the fibers are observed between 1640 and 1690 cm^−1^. The presence of bands in different fiber materials confirms the suitability for the formation of the composite with epoxy for effective use. Similarly, epoxy matrix functional groups can be probed or investigated with FTIR, and the possible interaction can be observed. A typical example of an epoxy–neem matrix was characterized by FTIR analysis with identifiable characteristics peaks. The presence of carbonyl bands representing eliminated acetic acid resulted in an acetoxy group (neem) of carbonyl, and an OH-group was observed at 1736 cm^−1^. Other notable bands, namely, O-H, C-H, and C-O, are observed at 2966 cm^−1^, 1507 cm^−1^, and 1295 cm^−1^, respectively, as shown in Figure 11 [174].

### 7.3. X-ray Photoelectron Spectroscopy (XPS)

X-ray photoelectron spectroscopy (XPS) analysis is an important characterization technique to obtain quantitative and chemical information about the composite or surface modification on the fiber in the process of composite formation. For instance, carbon fiber (CF) is usually modified with several nanoparticles (cellulose nanoparticles, graphene-based, etc.) to achieve good interphase and stronger adhesion between the fiber and epoxy during the formation of the composite [175]. Additionally, fiber coating (fiber sizing) ultimately improves wettability, stress transfer, and mechanical properties. Batista and Drzal [176] utilized cellulose nanocrystals (CNC) to modify CF, and the XPS analysis was used to confirm the formation of a new composite, while the hydrophobic nature of CNC in epoxy resin was overcome by the treatment with APTES.

### 7.4. Nuclear Magnetic Resonance (NMR)

**Figure 12:**
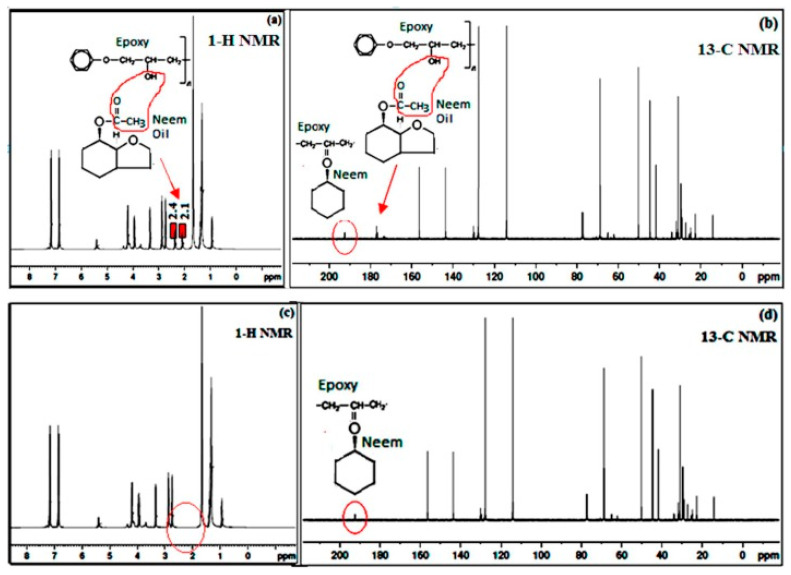
NMR: (**a**) 1 H NMR and (**b**) 13 C NMR analysis of epoxy–neem oil matrix at 80 °C, and (**c**) 1 H NMR and (**d**) 13 C NMR at 120 °C [177] (Reprinted/adapted with permission from Elsevier Ref. 5457240222636 Copyright 2018).

NMR analysis is a very powerful tool to primarily characterize the epoxy polymer resin and its composite for purity, the formation of a new matrix, and the identification of impurities. The use of 1-H (proton) and 13-C (carbon) NMR nuclei can be used to admire the matrix formation and the purity of the starting epoxy material. Furthermore, the presence of cellulose, hemicellulose, and lignin from the natural fiber sources can be identified through the NMR spectrum. For instance, the cellulose peak on 13-C NMR using CPMAS can be observed at 106 ppm, while peaks at 128 and 143 ppm are characteristic peaks associated with lignin [177]. The acetyl group of hemicellulose is observable at approximately 23 ppm for *furcraea foetida* fiber, and this observation was corroborated by NMR studies of natural fibers by other authors [178]. In the same vein, the purity of the epoxy–neem matrix was investigated by proton (1-H) and carbon (13-C) NMR for the composite heated at 120 °C [174]. The production of acetic acid when the heat treatment was applied confirms the purity of the epoxy–neem matrix. In the 1-H NMR analysis, the presence of CH_3_CO from the acetic acid group is noticeable at approximately 2.1 ppm and 2.4 ppm, while the peak of the hydroxyl group was suppressed due to low intensity before the heat treatment. The same observation was noted in the 13-C NMR, where peaks at 177 ppm and 192 ppm represent the acetic and ketone groups, respectively. Upon heat treatment at 120 °C, the loss of peaks at 2–2.7 ppm (from 1-H NMR) indicated the absence of an acetic group and was substantiated by the 13-C NMR without the peak at 177 ppm. The formation of a new matrix of epoxy-neem was established with the presence of a ketone group at 192 ppm and essentially shows that NMR provides useful information in the identification of structural components, as shown in Figure 12.

### 7.5. X-ray Diffraction

**Figure 13:**
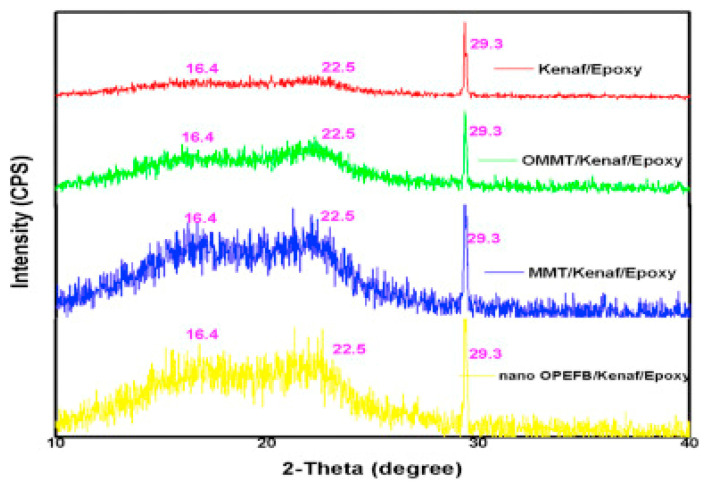
XRD-graph of kenaf/epoxy composites and filler-filled kenaf/epoxy hybrid nanocomposites [179] (Reprinted/adapted with permission from Elsevier Ref.501782938 Copyright 2018).

Crystalline and amorphous phases in the composite materials can be elucidated from X-ray diffraction (XRD) analysis. Similarly, the fiber-epoxy-related composite can be aptly studied by XRD. The structural crystallinity and phase identification of oil palm fiber (OPEFB), kenaf fiber, montmorillonite (MMT), organic-modified MMT, and epoxy were investigated by X-ray diffractograms [152]. The formation of the composite with homogeneous dispersion was confirmed for MMT/kenaf/epoxy, OMMT/kenaf/epoxy, and OPEFB/kenaf/epoxy. Furthermore, the absence of reflections between 0.4 and 10° indicates the formation of a nanocomposite (exfoliated) of lager d-spacing and the homogeneous dispersion of the epoxy-kenaf composite. However, the peak at 2-theta = 22.5° in the OMMT/kenaf/epoxy composite shows the presence of OMMT and the peak at 2-theta = 29.3° indicates the characteristics of kenaf fiber moieties, as shown in all composites (Figure 13) [179]. In essence, XRD is a non-destructive technique used to understand crystallography structure (crystalline or amorphous) concerning homogenous and heterogeneous composites for practical purposes.

## 8. Application of Non-Destructive Testing in Epoxy-Based Natural Fiber Polymer Composites

**Figure 14:**
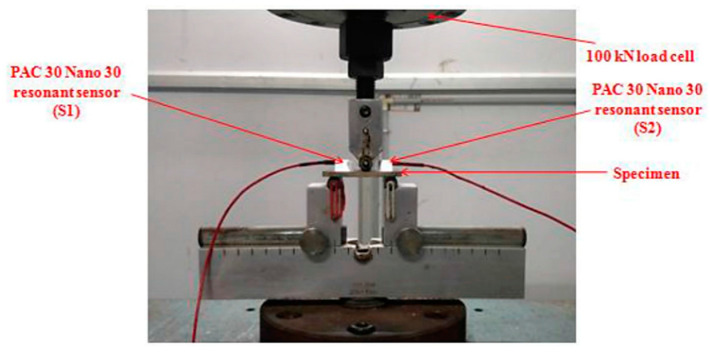
Use of AE data acquisition software when the specimen is subjected to flexural loading [181] (Reprinted/adapted with permission from Elsevier Ref.5457241208643 Copyright 2019).

The application of non-destructive testing has gained huge acceptance in polymer composites. Numerous research studies have applied the usage of non-destructive testing in evaluating the validity of test materials such as the one found in epoxy-based natural fiber polymer composites. Idolor et al. [180] used non-destructive testing to examine polymer composites by exploring the polymer–water interface and damage-dependent hysteresis. In this research, moisture, which served as an imaging agent, is naturally absorbed in the polymer composite for damage detection, and with the use of machine learning (logistic regression), damaged and undamaged regions were classified. Kumar et al. [181] used the acoustic emission (AE) technique to investigate the temperature and hybridization effect on the drop weight impact and post-impact residual strength of reinforced hemp and basalt polymer composite laminates. The composites at temperatures of 35 °C and 65 °C were first subjected to a drop weight impact, which was later followed by a three-point bending test to determine and record its residual strength and AE signals. With the use of AEWin and 8-channel AE data acquisition system software (shown in Figure 14), the AE output of the sloped total rise angle (RA) was used for determining the effect of impact-induced damage in the laminates. The recorded average velocity in hemp/epoxy, basalt/epoxy, and hybrid epoxy (hemp-basalt epoxy) was 3020 m/s, 3450 m/s, and 3200 m/s, respectively.

**Figure 15:**
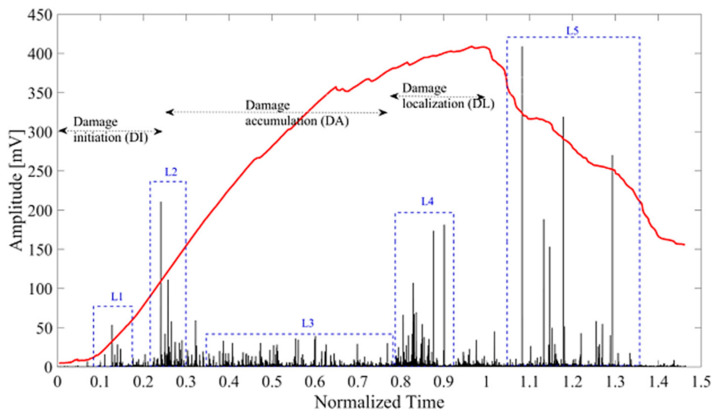
AE signal indicating the load vs. time [183] (Reprinted/adapted with permission from Elsevier Ref.5457241510623 Copyright 2021).

In conclusion, the AE in combination with the sentry function and total RA values were enough to detect the gradual failure and damage-tolerant nature of the laminates, which shows that the hybridized laminates have a better resistance against impact damage at a higher temperature when compared with non-hybridized (hemp/epoxy and basalt/epoxy) laminates. In a similar study conducted by Liu et al. [182], AE was used to study the failure mechanisms of fiber-epoxy laminates by setting up mapping between failure properties and AE features (energy, amplitude, etc.) while examining different lay-up patterns and hole sizes of laminates. Moreover, Friedrich et al. [183] used a low-frequency acquisition AE technique to determine the damage process in a glass-fiber-reinforced polymer (GFRP). Figure 15 shows the positive aspect of the AE signal with the plot of the load vs. time curve for the GFRP plate. Their approach aided the identification of AE in a semi-automatic manner with faster examination during the processing of data of global parameters.

Nsengiyumva et al. [184] conducted a comprehensive review of the advances, limitations, and non-destructive testing and evaluation (NDET) of thick composites and sandwich structures. They focused on the usage of NDET on composites greater than or equal to a 15 mm thickness while discussing flaw detection, localization, and characterization. It was concluded by [184] that despite increasing usage of NDET on composite materials, there have not been any real-time flaw size measurements. Moreover, little research has been performed on the application of NDT to hybrid reinforced composites of complex shapes and thinner sections. A review of the common NDET methods such as visual testing, electromagnetic testing, radiographic testing, thermography, acoustic emission, etc., on composite materials was carried out by Gholizadeh [185].

**Figure 16:**
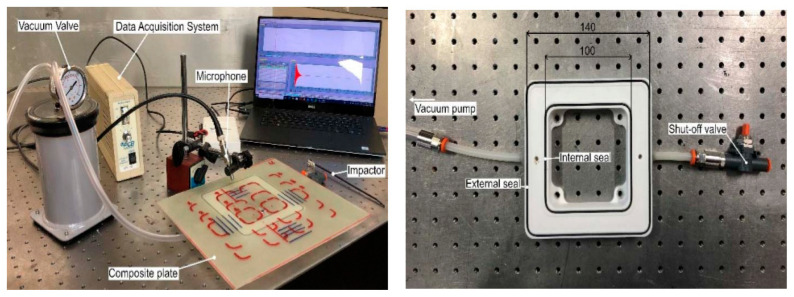
An experimental setup of IET method on a 3D-printed polyethyleneterephthalate (PTE) glass-fiber composite [186] (Reprinted/adapted with permission from Elsevier Ref.5457250276906 Copyright 2021).

Using the impulse excitation technique (IET), Niutta et al. [186] determined the local variation of the elastic properties of damaged regional composite components by measuring its vibrational response. They validated the IET method on two glass-fiber (six-layer and eight-layer composite laminates) woven fabric composites with epoxy resins. The setup is shown in Figure 16.

It was concluded that the residual elastic properties assessed by the IET method are in line with those obtained through the optic fiber method, therefore justifying the accuracy of the method. A study on the piezoelectric transducer-based (PTB) nondestructive testing method was carried out by Na [187]. He used electromechanical impedance (EMI) to differentiate the crack damage from the de-bonding damage of the GFRP plate. The data obtained from the variation in the impedance signature is combined with different methods of piezoelectric transducer attachment to achieve the differences between the crack damage case and the de-bonding damage case. In another report performed by Alves [188], nuclear magnetic resonance imaging (NMRI) was used to characterize artificially inhibited fractures (translaminar and delamination) of continuous fiber-reinforced polymer (CFRP) matrix composites laminates. The study established a background for in vivo use of this non-lethal NDET method to determine the level of the structural integrity of an advanced orthopedic implant while also estimating the lifespan of such implants. In earlier research conducted by Park et al. [189], acoustic emission (AE) testing was used alongside fragmentation tests to carry out an interfacial evaluation of electrodeposited single fiber/epoxy composites. Due to the principal failure around the fiber fracture, the pencil-lead-break method (an AE method) was used as a function of the epoxy matrix modulus and surface treatment by electrodeposition (ED) to measure the fiber fracture locations. It was concluded by Park et al. [189] that AE serves as a useful and dependable method to measure the polymer composites’ interfacial shear strength and mechanical properties.

### Biodegradability Studies of Epoxy-Based Natural Fiber Polymer Composite Materials

Biocomposites could be conceived as polymeric composites filled by natural fibers in different volume/weight concentrations, orientations, sizes, and shapes. Biocomposites are different from biopolymers as biopolymers are naturally biodegradable polymers and are produced by living organisms. The term biodegradable therefore implies the ability to break down and be absorbed into the body or system [190]. The synthesis of a bio-based epoxy polymer is strongly recommended to reduce the utilization of petroleum resources and subsequent emissions of CO_2_ [191]. The use of solvent and high temperatures for the chemical recyclability of epoxy resins has been demonstrated, but such practices restrict their potential utilization in natural fiber-reinforced vitrimers. The two common approaches used for evaluating and elucidating the biodegradability performances of natural fiber/polymer composite materials are the accelerated weathering test and the soil burial biodegradability test.

## 9. Accelerated Weathering Test

As a means of studying the degradation of composite materials, accelerated weathering testing is performed by utilizing the accelerated weathering tester. The test is usually performed following ASTM G154-00 where the specimens are exposed to fluorescent light with irradiation. Chee et al. [192] examined the degradation of bamboo/kenaf/epoxy hybrid biocomposites using accelerated weathering testing. The specimens were irradiated with irradiation of 0.77 W/m^2^/nm for 8 h at 60 °C followed by 4 h of condensation at 50 °C. All the samples were tested for a period of 156 h. The changes in the color of weathered and unweathered samples were compared by visual inspection of the photographic images. The total color changes (ΔE) were evaluated using Equation (9):

(9) $$\Delta E=\sqrt{{\left(\Delta L\right)}^{2}+{\left(\Delta a\right)}^{2}+{\left(\Delta b\right)}^{2}}$$

where ΔL, Δa, and Δb denote the differences between the initial unweathered sample and the final weathered sample.

**Figure 17:**
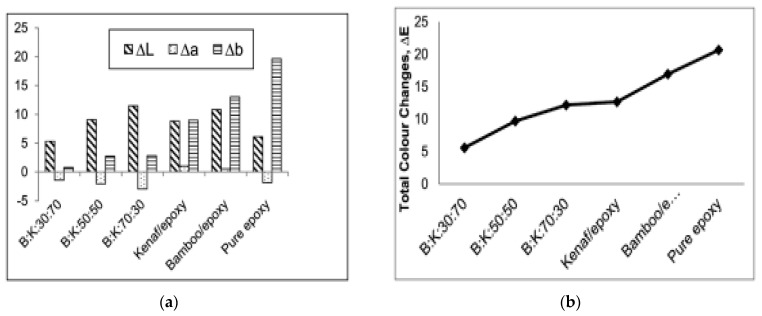
(**a**) Change in brightness $\left(\Delta L\right)$ and chromatic coordinates (Δa, and Δb ), (**b**) total change in color after the samples have been exposed to weathering process [192] (Reprinted/adapted with permission from Elsevier Ref.5457250582417 Copyright 2019).

It was established that pure epoxy showed prominent color changes and was closely followed by the bamboo/epoxy composite. Of all the hybrid composites examined, 30 wt% bamboo and 70 wt% kenaf turn the lightest color, followed by 50 wt% bamboo and 50 wt% kenaf, and then 70 wt% bamboo and 30 wt% kenaf, which displayed a dark and brownish appearance. The kenaf/epoxy and bamboo/epoxy composites displayed parity in the color changes of the components (ΔL and Δb); however, minimal changes in the red/green component (Δa) were observed, as shown in Figure 17a. However, several researchers [193,194] have opined that pure epoxy changes from transparent to yellowish, and this is attributed to the degradation of chromospheres that forme during polymer fabrication. In addition, the total color changes concerning various composite samples are presented in Figure 17b. As evident in Figure 17b, increasing the weight fraction of bamboo fiber results in a higher intensity of color changes, and this is caused by a high content of lignin in bamboo fibers compared to kenaf fibers. Many reports have shown that high lignin content in biocomposites promoted discoloration when subjected to accelerated weathering [195,196,197]. Thus, the ΔE for all the hybrid composites is lower than the control samples, and this explains the effect of hybridization.

### Soil Burial Biodegradability Test

**Figure 18:**
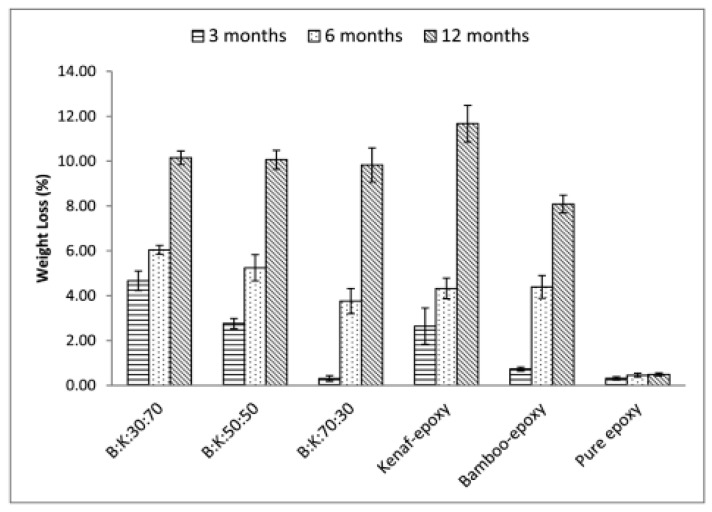
Weight loss of the composites for periods of 3, 6, and 12 months [192] (Reprinted/adapted with permission from Elsevier Ref.5457250582417 Copyright 2019).

Biodegradability tesingt of epoxy/natural fiber composites is possible through a soil burial test. Biodegradation occurs because of the moisture and enzymatic action of the microorganisms with consequential loss in the weight of the composites. The test is usually performed following the procedure of Obasi et al. [198]. Chee et al. [192] carried out soil burial tests of epoxy-containing bamboo and kenaf fibers for periods of 3, 6, and 12 months. The samples were cut to specific dimensions and then buried in wet soil in a perforated polybag, and biodegradability was evaluated from the weight loss of the specimen for different periods. After the time had elapsed, the samples were removed, washed with distilled water, and thereafter dried in an oven for 24 h. at 70 °C. The weight loss was calculated using the Equation (10):

(10) $$W_{loss}=\left(W_{initial-}W_{final}\right)/W_{final}\times 100$$

where $W_{initial\;}\mathrm{and}\;W_{final}$ are the weights before and after the test, respectively. As evident in Figure 18, the weight loss of the samples became significant with increasing burial time. Increasing the kenaf fiber fraction promoted weight loss for the period of 3 and 6 months. Thus, 30 wt% bamboo and 70 wt% kenaf epoxy hybrid composites exhibited the highest biodegradability (weight loss). It is important to state that increasing the hygroscopic characteristics of the material enhances microbial activity, which resulted in weight loss [199]. As reported in the literature [200], the hemicellulose component of the fibers is the motive behind moisture absorption and biodegradation of the composites.

Furthermore, Mittal and Chaudhary [201] investigated the biodegradability of pineapple leaf/coir fiber epoxy hybrid composites after processing through the hand lay-up method. Both treated and untreated forms of the fiber were used in the composite preparation and the samples were buried in the soil for 110 days. Every 10 days, the samples were removed from the soil, washed with distilled water, and then dried in an oven at 70 °C for 24 h. The loss in weight of the samples was evaluated, and it was revealed that the pineapple leaf/epoxy composite has the maximum weight loss compared to other samples, and this was attributed to the high hemicellulose content of pineapple leaf fiber. It was equally observed that the weight loss of the hybrid composite is reduced with an increase in coir content, and this was attributed to the high lignin content of coir fiber that resists microorganism attack. Moreover, the untreated fiber epoxy composite showed a higher weight loss as compared to its treated counterparts. Treated fiber epoxy composites are expected to exhibit a higher rate of biodegradation. The removal of OH and other polar functional groups from the fiber following treatment was reported to have been responsible for the low weight loss of treated fiber epoxy composites. Moreover, the mechanical properties of the composite samples were found to decrease drastically after the biodegradation test.

Investigation on the biodegradable performances of Emu feather/epoxy composites was presented by Chandrasekhar et al. [202]. Emu feather was first treated with sodium hydroxide prior to their utilization in the reinforcement of the epoxy matrix. The fabricated Emu feather/epoxy composites were exposed to the atmosphere in three different locations. Furthermore, the composites were buried beneath the earth above the three locations specified earlier. To evaluate the weight loss of the samples, they were pre-weighed and then weighed after one month of exposure in each case. The experiment was performed for three months; however, each month, the samples were cleaned with distilled water and then dried. For the samples exposed to the atmosphere, there was a significant loss in weight and thickness after the first two months. Thereafter, there was no significant change in either the weight or thickness of the samples. However, for the samples buried in soil, there was weight gain for the first two months, and thereafter, it remained constant. Thus, the weight gain of the samples buried in soil was attributed to moisture absorption, which increased their weight. Dinesh et al. [23] equally examined the biodegradation of epoxy filled with wood dust and jute fibers after fabrication by the hand lay-up technique. Biodegradation of the hybrid composites was observed through weight loss after being buried in soil for a period of 50 days. It was observed that samples without wood dust showed the highest biodegradability as jute fiber exhibited the highest tendency to absorb moisture from the composite and thus accelerated biodegradation [203].

It is therefore imperative to state that the degradation of epoxy/natural fibers biocomposites involves the breakdown of composite materials with the loss of mechanical properties [204]. This occurs through the breakdown of cellulose, hemicellulose, and lignin components of the reinforcement fibers. The decomposition of these components of the fibers is catalyzed by atmospheric moisture, temperature, pressure, ultraviolet light, and the action of the microorganisms. It is clear that natural fibers in epoxy matrix enhance their biodegradation; however, the degree or extent of biodegradability of the biocomposites depends on the fiber types. Fibers with a high hemicellulose content are more biodegradable than those with lower hemicellulose concentrations.

## 10. Application of Epoxy-Based Natural Fibers Biocomposite Materials

The interesting properties of an epoxy matrix, which include excellent damage tolerance, remarkable mechanical properties, perfect dimensional stability, high chemical resistance, and good electrical and thermal insulation performance, have made it a competitive polymer matrix material. Moreover, the density of natural fibers, which is less than synthetic man-made fibers, has made natural fibers suitable for lightweight reinforcement in the production of lightweight composite materials [177]. Currently, natural fiber epoxy biocomposite materials are used in different fields of applications, including the automotive industry (door panels, engine and transmission covers, seat back rests, and underbody panels). Some parts of automobiles such as the engine hoods, dashboards, and storage tanks are made using natural fiber reinforcement such as flax, hemp, jute, sisal, and ramie [15,94,120,153,171]. The automobile sector specifically has shown a genuine commitment to economic and environmental concerns by using natural fibers in different non-structural components with the goal of lowering mass, fuel consumption, and emissions [123].

Regarding aerospace applications, fiber-reinforced epoxy composites with unique properties are required for aircraft interiors. Fiber-reinforced epoxy composites show numerous applications in the aerospace industry due to their superior mechanical properties and lightweight structure [123].

Regarding Marine applications, fiber-reinforced epoxy composites have shown potential benefits of replacing various components such as ship hulls, propeller blades, and wind and tidal turbine blades [93]. Oil and gas applications include the production of underground pipes, boat building, etc. [39]. Some specific composites developed for certain special applications are reported below.

Sumesh et al. [171] developed an epoxy-based bicomposite by utilizing pineapple, banana, coir fiber pineapple ash, banana fiber ash, and coir fiber ash as fillers. With increasing silica content from the ash, the biocomposite mechanical properties were improved, and such lightweight materials are highly desirable in lightweight automobile applications. Moreover, Rajeshkumar et al. [55] fabricated sodium hydroxide-treated Phoenix. Sp fiber/epoxy composites. The fiber treated at a 15% concentration NaOH solution had the best impact properties and could be used for developing automotive panels and other industrial applications requiring a high structural impact. Furthermore, bacterial cellulose (from coconut fibers)/Kevlar hybrid epoxy composites were developed by Rusdi et al. [205]. The composites were found to have exceptional strength and impact energy, and such composites are good candidate materials in marine and bullet applications. In the area of thermal insulation, epoxy-based biocomposites are equally valuable thermal insulators. For example, Chowdari et al. [35] fabricated areca/coconut shell powder epoxy hybrid composites, and the thermal properties of the epoxy were found to decrease with increasing fiber contents. The hybrid composite material was found to be an excellent thermal insulator. Regarding ballistic applications, hemp fiber/epoxy composites have been reported to be an excellent candidate material, especially composites containing 20–30 wt% hemp fibers in the epoxy matrix [159]. In non-structural and building material applications, Jawaid et al. [10] developed hybrid date fiber/bamboo epoxy composites with interesting mechanical properties and thermal stability. Such materials are good in the building and construction industry. Regarding sound absorption materials in ceiling and wall applications, Arumugam et al. [33] developed rice husk and saw dust epoxy biocomposites materials, which were found to have good mechanical properties, thermal stability, high electrical resistivity, and excellent acoustic absorption properties.

Other applications of natural fiber-reinforced epoxy composites include civil and structural applications, which involve the construction of new advanced structures (roofs, plate, and shell elements, linear elements, pipes and tanks, folded structures, and so on), as well as the rehabilitation of existing metallic and concrete structures such as buildings, bridges, pipelines, masonry construction, etc.; sporting applications such as golf club shafts, tennis rackets, bicycle frames, and fishing rods; electrical and electronics component applications including powerline insulators, fiber optics, tensile members, and lightning poles. Chemical industry applications include stacked bottles for fire departments, composite containers for substances, mountain climbing, ducts and stacks, and subterranean storage tanks. Medical field applications of natural fiber-reinforced epoxy composites include orthopedic medicine, prosthetic devices, and imaging [32].

Understanding the significant material characteristics of fiber/epoxy constituents, as well as the fundamental structures and availability of production technologies, is required for the use of fiber/epoxy composites in a range of applications. The manufacturing technique used has an effect on the ultimate quality of the material. The cost of materials is influenced by the production volume—the bigger the volume of production, the lower the cost of materials.

## 11. Future Directions

The production of biobased polymer composite materials with huge environmental compliance is a synergistic effort of scientists, engineers, and industrialists. Natural fiber/epoxy biocomposite materials have proven to be candidate materials with interesting properties. However, the lack of compatibility of natural fibers with the epoxy matrix and the water absorption of natural fibers militates against their commercial success. The treatment of natural fibers prior to their inclusion has been suggested by researchers, but the optimization of the treatment process and the appropriate treatment method is a subject of debate. In the current state, alkaline treatment and hot water treatment are conceived to be promising treatment techniques for possible commercialization. Therefore, these two methods need to be investigated on the available natural fibers, and the treatment parameters need to be optimized for economic purposes to reduce the overall cost of biocomposite production.

Moreover, natural fiber/epoxy polymer composite materials may not meet the property requirements as compared to synthetic fiber epoxy composites. The mechanical properties of epoxy/synthetic fibers are still higher than epoxy/natural fiber composites. Hybridization of natural fibers with synthetic fibers could provide a means of combining the advantages of natural and synthetic fibers in the epoxy matrix. Such hybrid epoxy/fiber composites are expected to meet the demand of the current industries, and researchers are therefore encouraged to devote significant attention to the development of hybrid natural fiber epoxy biocomposite materials with adequate mechanical properties, physical properties, and biodegradability.

## 12. Conclusions

Natural fibers are extensively used in the reinforcement of the epoxy matrix because of their ability to make a strong bond with the matrix. In comparison to synthetic fibers, natural fibers are easy to process, low cost, and available in abundance and have appreciable thermal properties and good mechanical properties. Generally, natural fibers are obtained from plant and animal resources. Common fibers obtained from plants include jute, coir, pineapple peel, banana peel, sisal, jute, kenaf, and date palm while wool and silk are the two most common fibers from animal resources. Interestingly, animal fibers have high strength but low stiffness and are more expensive compared to plant fibers.

The production of biocomposite epoxy-based natural fiber materials with tailored properties is of the utmost concern for scientists and industrialists. The mechanical properties of epoxy-based natural fiber composites depend on the nature of the fiber, volume fraction, orientation and surface treatment, and production method employed. Various fiber treatment options were mentioned in this work, and alkali treatment is adjudged to be the best treatment method in terms of its effectiveness and economy. Mechanical properties such as tensile strength, flexural strength, impact strength, and hardness rely on the strength of the interfacial adhesion between the fiber and the matrix. Other properties of the epoxy matrix such as thermal properties, electrical properties, water absorption, and acoustic performance all vary with natural fiber loadings. To understand the nature of the interfacial adhesion between the fiber and the epoxy matrix, characterization tools such as SEM, TEM, and XRD are necessary. Other various characterization tools such as DSC, NMR, and FTIR are clearly described in this work. Two means of investigating the biodegradability study of epoxy-based natural fiber polymer composites (accelerated weathering and soil burial test) are clearly presented and described extensively in the current study. Applications of epoxy natural fiber biocomposites in different fields such as automotive, aerospace, biomedical, buildings, and thermal insulations, etc., are explored. Optimization in natural fiber treatment and the hybridization of natural fibers with synthetic fibers in epoxy matrix composites are future research directions.

## Figures and Tables

**Table 1:** Mechanical properties of epoxy-based natural fibers and glass hybrid polymer composites.

Fiber	Fiber Content (wt%)	Tensile Strength (MPa)	Elastic Modulus (GPa)	Elongation at Break (%)	Flexural Strength (MPa)	Flexural Modulus (GPa)	Impact Energy (KJ/m^2^)	Hardness	Ref.
Abaca	2.6–7.9	21.67–29.87		2.14	19.07				[95]
Abaca + glass	40	44.5	0.27	15.05	12.5				[96]
Alfa	10	3.89–28.01	3.92–4.62	0.09–0.6					[8]
Bagasse	30	20.43–42.86	1.33–1.54	1.4–2.5					[97]
Bagasse + glass	15–30	1.56–4.69	20.5–56.7	20.5–56.7	0.82–3.96			39.80–53 HRA	[98]
Bamboo	64	87–135	6.74–8.2		92–154	5.66–11.9	8.95–30.25		[99]
Bamboo + glass	10	92–100	3.7–5.4		145–165	5.6–7		23.4–25.3 HV	[100]
Banana	25	16–35	0.5–1.7		35–68	7.8–13	2–12 (J/m)		[101]
Banana + glass	10–40	45–135	0.44–1.38	10.5–13	30–200				[102]
Coir	30	3.21–13.05	1.33–2.06		25.41–35.42		16–17.5	15–16.9 HV	[103]
Coir + glass	5–10	13.5–18	1.2–1.85		40–65			10–23 HV	[104]
Curaua	30–50	20.2–30.29	2.27–4.24	1.4–2	52.02–67.45	2.94–4.37			[105]
Date Palm	40–60	21.43–24.35	0.61–1.32	1.17–1.35			68.13–98.71 (J/m)		[106]
Flax	40	4.5–133	2.7–28		8–218	0.36–18			[107]
Flax + glass	16.5	225	9.06	5	170–420	4.2–14.6			[108]
Hemp	40–65	65–162	0.008–0.018		145–180	8–10.5	7–15		[109]
Hemp + glass	11–28	21.79–23.86	0.143–0.222	9–15	47.77–61.54	87.03–100.2			[110]
Henequen	64	233–234	15–17	0.9–1.7	197.32–199.52	11.90–12.23	90.81–116.04		[111]
Henequen + glass	10–50	60.8–156.2	2.3–3.2	2.7–5.3	80.65–128.6	7.0–11.4			[112]
Jute	30	78.21	1.52		203.48	9.76	5.67–13.89		[113]
Jute + glass	8.75	80–85	2.6–4.8		93–165	4.6–6.6			[114]
Kenaf	50	138.92–159.98	5.77–8.2	1.8–2.4					[115]
Kenaf + glass	14	150	7.5		220–240	3.87–14.2			[71]
Oil Palm	5–20	28–30	1.34–1.44		40–56				[116]
PALF	5–25		2.13–3.44			4.4–6.25	22–32.5 (J/m)		[117]
PALF + glass	10–20	38–48	1.2–1.75		88–152	3.5–7	3.5–7		[118]
Piassava	10–40	58.97–89.33	2.46–2.87		39.35–78.41	2.56–3.46	75–210 (J/m)		[119]
Ramie	42–49	85–117	8.3–11.8		130–152	7–9.5			[120]
Sisal	50–58	240–375	4–9.75	3–5.25	200–325	15–24			[121]
Sisal + glass	15–35	53.77–72.13			80.2–87		27.13–48.59		[122]

## Data Availability

The data presented in this study are available on request from the corresponding author.
